# *Hypericum perforatum* L.-Mediated Green Synthesis of Silver Nanoparticles Exhibiting Antioxidant and Anticancer Activities

**DOI:** 10.3390/nano11020487

**Published:** 2021-02-14

**Authors:** Abdalrahim Alahmad, Armin Feldhoff, Nadja C. Bigall, Pascal Rusch, Thomas Scheper, Johanna-Gabriela Walter

**Affiliations:** 1 Institut für Technische Chemie, Leibniz Universität Hannover, 30167 Lower Saxony, Germany; scheper@iftc.uni-hannover.de; 2 Institut für Physikalische Chemie und Elektrochemie, Leibniz Universität Hannover, 30167 Lower Saxony, Germany; armin.feldhoff@pci.uni-hannover.de (A.F.); nadja.bigall@pci.uni-hannover.de (N.C.B.); pascal.rusch@pci.uni-hannover.de (P.R.)

**Keywords:** *Hypericum perforatum* L. (St John’s wort), silver nanoparticles (AgNPs), mechanism of green formation of nanoparticles, DPPH, ABTS, antioxidant and cytotoxicity effects

## Abstract

This contribution focuses on the green synthesis of silver nanoparticles (AgNPs) with a size < 100 nm for potential medical applications by using silver nitrate solution and *Hypericum Perforatum* L. (St John’s wort) aqueous extracts. Various synthesis methods were used and compared with regard to their yield and quality of obtained AgNPs. Monodisperse spherical nanoparticles were generated with a size of approximately 20 to 50 nm as elucidated by different techniques (SEM, TEM). XRD measurements showed that metallic silver was formed and the particles possess a face-centered cubic structure (fcc). SEM images and FTIR spectra revealed that the AgNPs are covered by a protective surface layer composed of organic components originating from the plant extract. Ultraviolet-visible spectroscopy, dynamic light scattering, and zeta potential were also measured for biologically synthesized AgNPs. A potential mechanism of reducing silver ions to silver metal and protecting it in the nanoscale form has been proposed based on the obtained results. Moreover, the AgNPs prepared in the present study have been shown to exhibit a high antioxidant activity for 2, 2′-azino-bis-(3-ethylbenzothiazoline-6-sulfonic acid) radical cation, and super oxide anion radical and 2,2-diphenyl-1-picrylhydrazyl. Synthesized AgNPs showed high cytotoxicity by inhibiting cell viability for Hela, Hep G2, and A549 cells.

## 1. Introduction

Over the past decades, silver nanoparticles (AgNPs) have received considerable research interest, due to their unique chemical and physical features, and promising usages [1,2,3,4]. The use of environmentally benign materials to synthesize nanoparticles provides many benefits including eco-friendliness and compatibility to pharmaceutical and biomedical applications because of the avoidance of toxic chemicals in the synthesis protocols. Green synthetic methods for nanoparticles include biological methods, the use of polysaccharides, irradiation, polyoxometalates, and the Tollens method [5]. 

Biological methods include the use of algae to synthesize AgNPs at room temperature [6]; moreover several microorganisms (diatoms, fungi, bacteria) are utilized to grow AgNPs intracellularly or extracellularly such as colonic flora *Klebsiella pneumonia*, *Escherichia coli*, *Enterobacter cloacae*, *Bacillus subtilis*, *Penicillium*, *Vericillum*, *Fusarium oxysporum*, *Pseudomonas stutzeri*, *Phanerochaete chrysosporium*, *Penicillium fellutanum*, and other [7,8,9,10,11,12,13,14,15,16,17,18,19]. An exceptional work has been done in order to synthesize AgNPs using diverse biological regimes which involve extracts from plants such as *Carica papaya*, *Ocimum*, *Capsicum annuum*, leaves of *Azadirachta indica*, as well as many other plants [20,21,22,23,24,25,26,27,28,29,30,31,32,33,34,35,36,37]. The bioreduction of silver ions to AgNPs occurs due to the presence of biomolecules that are found in plant extracts, such as flavonoids, terpenoids, phenolic acid, alkaloids [38]. The synthesis of nanoparticles through the use of plant extracts attracts a lot of attention, mainly due to its simple methodology which does not require a sophisticated process such as maintenance of microbial cultures and multiple purification steps. Moreover, the biosynthesis can be adjusted to customize the size and shape of nanoparticles by using various amounts of plant extract and metal ions in the reaction medium [39,40]. The biosynthesis of AgNPs is a complex approach, which is not fully addressed yet [41]. AgNPs are increasingly used in various fields because of their unique physical and chemical properties. These include thermal, electrical, optical, high electrical conductivity, and biological properties [12,42,43]. Because of their special characteristics, they have been used for several applications, including in medical device coatings, as antibacterial agents, as additives in cosmetics, and in the pharmaceutical industry, within the food industry, orthopedics in diagnostics, in drug delivery, and as anticancer agents, where they enhance the effects of anti-cancer drugs [44]. Moreover, silver nanostructures were employed for treatment of burns and wounds, for impregnating textile fabrics and as well as a contraceptive and marketed as a disinfectant for water [45,46,47,48], antibacterial for both, Gram-positive and Gram-negative bacteria [49,50,51,52,53], antiviral [54,55,56], in the electronics and optoelectronic devices [57,58,59], antimicrobial and antifungal agents [60,61,62], as optical labels such as in optical sensors [63,64,65], catalysts [66,67], drug delivery systems [68,69], and in the field of cancer treatment [70,71,72,73]. 

*Hypericum perforatum* Linn., generally recognized as St. John’s Wort is a flowering plant and is native in Asia and Europe. It belongs to the Hypericaceae family that contains more than 1000 species and about 55 genera. *Hypericum* genus possesses more than 450 species distributed worldwide in tropical and subtropical regions [74]. *Hypericum perforatum* (St. John’s wort) has been used as traditional medicinal plant all over the world [75], due to its large diversity of secondary metabolites with considerable pharmaceutical effects [76]. *Hypericum perforatum* Linn. has been proven to be potential in treatment of many different diseases like cancer, AIDS, and depression [77,78,79,80]. *H. perforatum* exhibits analgesic, anti-inflammatory, antioxidant, anticonvulsant, antidiabetic, and cytotoxic activities [81,82,83]. The significance of its most important constituents like hypericin, hyperforin, flavonoids and their analogs is attributed to their botanical safety and therapeutic efficacy [84]. Hypericin is renowned as a photosensitizing agent employed in the photodynamic therapy of viral infections and cancer. Lavie et al. [85] demonstrated the inhibitory influence of pseudohypericin and hypericin against influenza virus, herpes simplex virus types II and I, and vesicular stomatitis. Lopez-Bazzocchi et al. [86] and Hudson et al. [87] investigated the deactivation of sindbis virus, HIV-I, and murine cytomegalovirus by treatment with hypericin and exposure to fluorescent light. Both pseudohypericin and hypericin block the viruses by production of singlet oxygen upon light irradiation [88].

In this work, we report on the single step facile synthesis of highly water dispersible AgNPs using aqueous extract of *Hypericum perforatum* Linn. To obtain AgNPs with desired characteristics, such as small size and narrow size distribution, and high colloidal stability with high yields, various protocols for the synthesis of AgNPs using plant extracts were used and compared. Originally these protocols (Table 1) were established from different authors using extracts of various different plants. Here we have used them to identify the protocol resulting in highest quality of AgNPs with characteristics beneficial for drug delivery applications, in particular a size between 10 and 50 nm, when using aqueous extracts of *Hypericum perforatum* L. Resulting AgNPs were thoroughly characterized with regard to size and stability, as well as with regard to the nature of the capping agent on the surface of the particles. 

## 2. Materials and Methods

### 2.1. Materials 

Aerial parts of *Hypericum perforatum* L. (St. John’s wort) were collected in June–July in southeastern Syria during the flowering season. Silver nitrate; 1,1-diphenyl-2-picrylhydrazyl (DPPH); potassium persulphate; 2,2′-azino-bis-(3-ethylbenzothiazoline-6-sulfonic acid) (ABTS) were purchased from Sigma-Aldrich (Darmstadt, Germany), Filter paper Whatman 90 mm from GE Healthcare Life Sciences (Freiburg, Germany), 0.22 µm nylon syringe Filter and Sartolab Vakuumfilter 180C5; 0.22 µm Polyethersulfon, 500 mL; syringe filter 25 mm, 0.45 um RC with GF prefilter; 0.45 μm PTFE filter and Vivaspin 10 kDa from Sartorius (Goettingen, Germany). Ethanol, methanol, and acetone were of HPLC grade from Roth (Karlsruhe, Germany), and acetonitrile from VWR (Hannover, Germany). Water was purified by a QM system from Sartorius (Goettingen, Germany).

### 2.2. Preparation of Hypericum perforatum L. (St John’s Wort) Extract

Aerial parts of St John’s wort were collected from the Ghab Plain, Syria in June and July. The plant was washed several times to remove dust and possible sludge. The samples were dried at room temperature in the dark and ground in an electric grinder. The powder (350 mg) was added to 800 mL of deionized water in a 1000 mL Erlenmeyer flask. The mixture was heated to maintain a gentle boiling for 4 h until the volume was reduced to approx. 250 mL. The extract was cooled to room temperature then clarified using Whatman filter paper (No. 40). The extract was centrifuged at 23,015× *g* for 30 min, and then filtered through a 0.2 micron filter. The resulting extract was stored in the refrigerator at 4 °C to be used directly in preparing AgNPs or freeze-dried (solvent was evaporated under nitrogen gas at 30 °C to prevent oxidation). This resulted in a yellow-brown solid with a yield of 19%. 

### 2.3. Biosynthesis of Silver Nanoparticles

**Figure 1:**
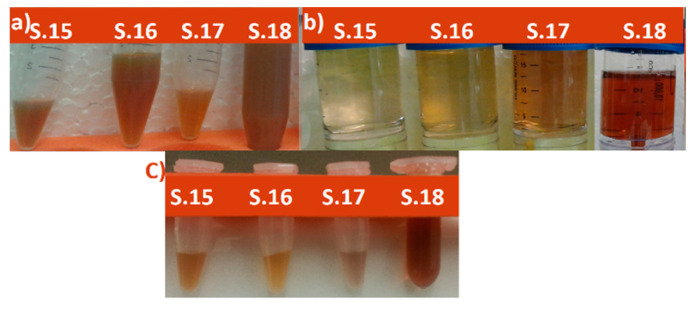
Images of the some samples (**a**) immediately after preparation (**b**) when washed using Vivaspin 10 kDa and (**c**) after washing. S mean sample Number.

Aqueous St John’s wort extract were mixed with silver nitrate and placed on magnetic stirrer with heating for a specific time (details given in Table 1) to obtain silver nanoparticles (AgNPs). St John’s wort extract was used as a source of biological reducing and capping agents. The influence of several parameters like concentration and the amount of silver nitrate used, amount of the plant extract used, and the reaction temperature, was investigated by conducting various experiential trials. Various protocols were employed to prepare AgNPs using plant extracts to identify the most appropriate protocol. In fact, most of the reaction times and conditions (proportion of silver ions and St. John’s Wort extract) in Table 1 are obtained from the literature where other groups prepared silver nanostructures using other plant extracts. The goal was to select the protocol that resulted in the best quality and yield of AgNPs when using *Hypericum Perforatum* L extract. The methods are summarized in Table 1, more detailed experimental procedures can be found in the supplementary materials. After obtaining the AgNPs, the particles were washed with deionized water by using centrifugal concentrators (Vivaspin 10 kDa) for at least ten times, to remove the residual organic compounds (Figure 1).

### 2.4. 1,1-Diphenyl-2-picryl Hydrazyl (DPPH) Assay

Several aqueous solutions of AgNPs (10–400 μg/mL in deionized water) were prepared. Total of 100 µL of each solution was added to 300 µL of 0.004% ethanolic DPPH free radical solution and incubated for 30 min at room temperature under shaking condition. The absorbance was measured by a UV-VIS spectrophotometer (Thermo Fisher Scientific, Schwerte, Germany) at 517 nm; afterwards the results were compared with the corresponding absorbance of standard ascorbic acid concentrations (10–400 μg/mL).

### 2.5. 2,2′-Azino-bis-(3-ethylbenzothiazoline-6-sulfonic Acid Radical Cation) (ABTS) Assay

ABTS solution (7 mM in water) was mixed with 2.45 mM potassium persulphate in a ratio of 1:1 (*v*/*v*). The mixture was placed in the dark at room temperature for 18 h. The ABTS•+ solution was diluted about 20 times with water to reach an absorbance of 0.850 ± 0.05 at 734 nm. About 150 µL of the diluted ABTS•+ solution was added to 50 µL of different concentrations of the AgNPs and incubated for 6 min at room temperature. For the control, 50 µL of deionized water was used in place of AgNPs. Ascorbic acid was used as a positive control. Absorbance at 734 nm was measured spectrophotometrically. The percentage of inhibition was calculated using the same formula as in DPPH assay and the radical scavenging activity of AgNPs is expressed as the IC50 value.

### 2.6. Super Oxide Anion Radical (SO) Assay

The reaction mixture consisted of 50 µL Tris–HCl buffer (16 mM, pH 8.0), 50 µL NBT (nitroblue tetrazolium; 0.3 mM), 50 µL NADH (β-nicotinamide adenine dinucleotide, reduced disodium salt hydrate; 0.936 mM) and 100 µL of various concentrations of AgNPs in water. The reaction was initiated by adding 50 µL of PMS (phenazine methosulfate) solution (0.12 mM) to the mixture. The dissolved oxygen from the PMS/NADH coupling reaction reduces NBT to produce the superoxide anion. The reaction mixture was incubated at 25 °C for 5 min and the absorbance was measured at 560 nm against a blank sample. Gallic acid was used as a positive control and every sample was measured in triplicate to calculate the mean values and standard errors of mean (SEMs). By comparing the results of the test and the control, the percentage of inhibition was determined.

### 2.7. Cell Culture and Estimation of In Vitro Cytotoxicity of AgNPs

For cell culture experiments, HeLa, HepG2, and A549 (CLS Cell Lines Service GmbH, Eppelheim, Germany) cells were seeded and grown in Dulbecco’s modified Eagle medium (DMEM) (Merck KGaA, Darmstadt, Germany) supplemented with 10% fetal calf serum (FCS) (Merck KGaA, Darmstadt, Germany) and 1% penicillin streptomycin (PS) at 37 °C in 95% O_2_ and 5% CO_2_. The cells were sub-cultivated, when the confluence reached almost 80%. The number of cells was calculated employing hemocytometer and suspended trypsinized cells. For Cell Titer Blue (CTB, Promega, Germany) cell viability test, cell suspension with concentration of 8 × 104 cell/mL was prepared and 100 µL of suspension was added to every well of the 96-well plate (VWR, Hannover, Germany), corresponding to 8000 cells per well. Plates were incubated at cell culture conditions as stated above for 24 h, then medium was removed from wells and 100 µL of different concentrations (0, 0.35, 0.7, 1.4, 2.77, 5.54, 11.07, 22.14, 44.28, and 88.56 µg/mL) of AgNPs dispersed in medium were added (four replicates per concentration). Control contained no AgNPs. Plates were incubated at the same conditions as stated above for cell culture for 2, 5, 8, and 24 h for Hela and HepG2 and only 24 h for A549. After the incubation time specified for each sample, medium from control and AgNPs-containing media were removed, then 100 µL of prepared CTB solution (Promega, Cat. Number G8080, Walldorf, Germany) (1:10 (*v*/*v*) in basal medium) was added into every well, in order to indirectly evaluate cell viability (metabolic activity and relative cell number). As blank for the measurement, four completely empty wells were filled with Cell Titer Blue prepared solution, then the plates were incubated under the same conditions mentioned previously for one hour. Current fluorescence was measured in every well in the plates using microplate fluorometer (544Ex/590Em) (Fluoroskan Ascent, Thermo Electron Corp, Waltham, MA, USA). The fluorescence of blank was subtracted from fluorescence of every concentration then relative cell viability was expressed as a percentage to positive control. The relative cell viability for every sample was commensurate with values of fluorescence and was calculated according to the following formula:

$$\mathrm{Relative}\;\mathrm{cell}\;\mathrm{viability}\;\left(\%\right)=\frac{\mathrm{Fluorescence}\;\mathrm{of}\;\mathrm{sample}-\mathrm{Blank}}{\mathrm{Fluorescence}\;\mathrm{of}\;\mathrm{control}-\mathrm{Blank}}\;\times \;100$$

Origin (Version 8.5) was used to calculate the half maximal inhibitory value (IC_50_).

### 2.8. Characterization

The synthesis of the AgNPs in aqueous solution was monitored by recording the absorption spectra in a wavelength range of 300–700 nm using a Nano Drop ND1000 Spectrophotometer (Thermo Scientific, Wilmington, DE, USA). The hydrodynamic diameter of AgNPs was determined via DLS (Dynamic light scattering), and zeta potential and isoelectric point was measured using Zetasizer Nano ZS MPT-2 (Malvern, Worcs, United Kingdom) (see Figures S1 and S2 in the supplementary materials). For Fourier transformed infrared (FTIR) measurements, samples were analyzed on a Bruker FTIR Vertex 80 v spectrometer (Billerica, MA, USA) which was operated at a resolution of 2 cm^−1^, 32 scans in the region of 5000–370 cm^−1^, ATR type platinum diamante A225 with evacuated to less than 1 hPa or without evacuating. Scanning electron microscopy (SEM) and energy-dispersive x-ray spectroscopy (EDXS) analyses were made on a field-emission instrument of the type JEOL JSM-6700F (Tokyo, Japan), which was equipped with and EDX spectrometer of the type Oxford Instruments INCA 300. Secondary electron (SE) imaging and EDXS were made at an acceleration voltage of 0.5 kV and 15 kV, respectively. X-ray diffraction (XRD) was performed on a Bruker D8 Advance diffractometer (Karlsruhe, Germany) using Cu-Kα radiation (λ = 0.154178 nm). Diffractograms were recorded in the 2θ range between 15° and 110° in steps of 0.008 degrees per second. Specimens were prepared as a thin film by dropping an aqueous solution of AgNPs onto a <911>-cut polished silicon crystal as support. Then, specimen was dried slowly in air. Alternatively, powder that was received after freeze drying of AgNPs colloidal solution was used. Transmission electron microscopy (TEM) and EDXS, were performed using a field-emission instrument of the type JEOL JEM-2100F-UHR (Tokyo, Japan), which was operated at an acceleration voltage of 200 kV. The microscope was equipped with an EDX spectrometer of the sort Oxford tool INCA 200 TEM. TEM analyses were performed in both, the image mode (bright field (BF), dark field (DF), high resolution TEM (HRTEM)) and selected area electron diffraction (SAED) mode. The nonexistence of preferred orientation was checked in SAED of the sample by tilting the sample inside the mechanical border of the goniometer (±19 angle of inclination). In scanning TEM (STEM), high-angular dark-field mode was applied. We used Millipore water (resistance > 18.2 MΩ cm^−1^) (Sartorius, Goettingen, Germany) in all chemical reactions. Thermal gravimetric analysis (TGA) was done by using TGA/DSC 3+ from Mettler-Toledo (Giessen, Germany), from 25 to 1000 °C at a rate of 0.5 °C per minute with N_2_ gas flow.

## 3. Results and Discussion

### 3.1. UV-VIS Absorption Studies

**Figure 2:**
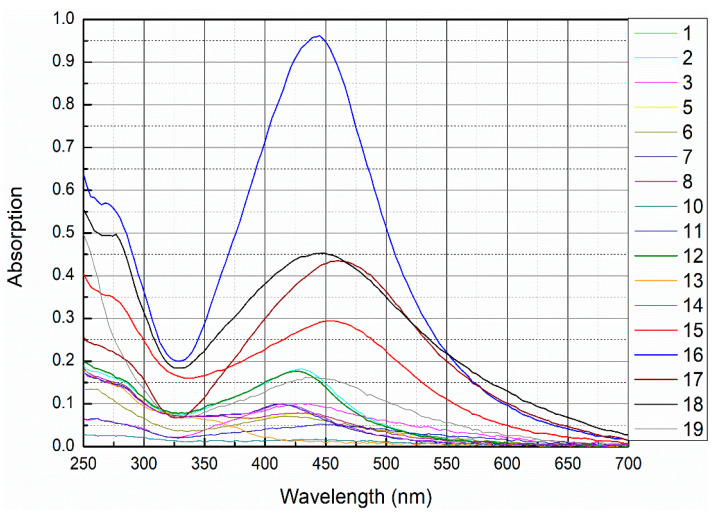
UV-VIS spectra of AgNPs synthesized with varying amounts of AgNO_3_ and different amounts of plant extract (numbers refer to the sample number shown in Table 1).

UV-VIS analysis is an important technique for verifying the formation of nanoparticles in colloidal solutions. Bioreduction of silver ions was visually clear from the color change. As St John’s wort aqueous extract was mixed with silver nitrate, the color changed from pale light to yellowish brown and the final color was reddish-brown, which indicates the formation of AgNPs [91]. Various colors were obtained (according to the concentration of AgNPs in solution and their size). The change of the color arises as a result of surface plasmon vibrations in AgNPs [25,92,93]. Figure 2 shows the UV–VIS spectra for the different samples obtained with different biosynthesis conditions as summarized in Table 1. The change of absorbance at λ_max_ is due to its dependence on the Ag NP concentration. The UV–VIS spectra showed the appearance of different absorption maxima between 392 nm and 460 nm, indicating the formation of silver particles with nanometer-sized dimensions. According to Figure 2, the peak for sample 16 (Table S1, supplementary materials) is sharp and high, while for some other samples (like 15, 17, 18, and 19) the peak is broad and less intense. Also there are broad peaks with low intensity (like for samples 1, 11, 3, and 14, Table S1, supplementary materials); also other peaks are barely detectable (like for samples 8, 10, 13, and 14). The broadening of peaks for some samples indicates that the nanoparticles were aggregated or the nanoparticles were polydisperse. The spectra of sample 16 shows a narrow SPR band with a maximum at 442 nm indicating the formation of uniform spherical nanoparticles (monodisperse AgNPs), which is further confirmed by the TEM images (Figure 6). After comparison of the different protocols summarized in Table 1 we found that protocols 15–20 gave the best results in UV-VIS, DLS and zeta potential but protocol 16 resulted in highest quality (in terms of size, shape and dispersion), yield, and purity of AgNPs, because other samples (15 and 17–20) contain other substances in addition to silver, such as calcium and silver oxide and yield of AgNPs was low compared to sample 16 as shown by XRD (Figure 10), EDX, and AAS analyses. Various temperatures have been investigated for protocol 16, plant extracts were heated to different temperatures (70, 80, and 91 °C) and silver nitrate was added to it, either at once or drop-wise. It was observed that at elevated temperature silver nanoparticles were produced faster, but particle size was bigger than for particles synthesized at 60 °C. 

#### Measurement of Concentration through Absorption Peak in UV-VIS Spectrum

Beer–Lambert Law was employed to calculate the AgNPs concentration as follows:

A=ε×l×c

*A* is absorbance, ε is size dependent molar extinction coefficient (M^−1^ cm^−1^), *c* is the concentration, and *l* is the length of the cuvette. The concentration was calculated by using the absorption values obtained for our different samples, L = 1.0 cm, and the values of ε molar absorption coefficient (extinction coefficient, molar Abs) were taken from reference [94], assuming that the nature of the protection layer on the surfaces of nanoparticles does not affect the value of this coefficient. All the details and results are found in Table S1 (supplementary materials).

Calculated concentrations were highest for samples 15, 16, 17, 18, 19, and 20. Consequently, all further studies were performed with samples which give high concentrations of AgNPs to identify the best synthesis in terms of size, size distribution, and quality of prepared nanoparticles.

### 3.2. Dynamic Light Scattering (DLS)

**Figure 3:**
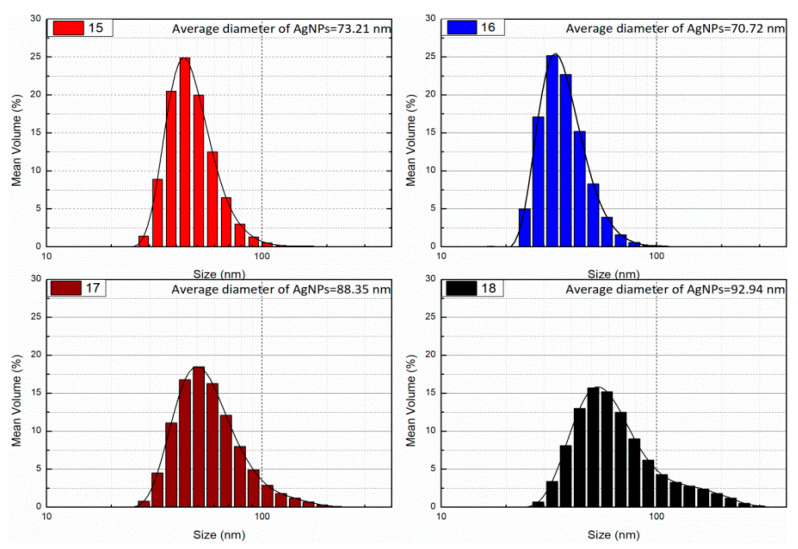
DLS analysis of AgNPs, hydrodynamic diameter of selected samples.

The size determined via DLS technique is the hydrodynamic diameter of the theoretical sphere, which diffuses at the same speed as the measured nanoparticle. Figure 3 shows the particle size distribution of the best samples of AgNPs. It was found that the AgNPs size was in the range of 44–112 nm. Hydrodynamic diameter for other samples are summarized in Table S2 (supplementary materials). A thin electric dipole layer of the solvent is adsorbed at the surface of nanoparticles when these particles are dispersed in solutions. This layer affects the motion of particles in the solutions, so the hydrodynamic diameter describes the metal core with any stabilizer substance and the solvent layer. Thus DLS measures the size of nanoparticles with the solvent molecules attached at its surface. In colloidal solutions when one layer of stabilizer (protective or capping agent) adsorbed at the surface of nanoparticles, the diameter increases, and commonly multiple layers of stabilizers and water molecules are present. Therefore, the protective layer and the interaction with the solvent molecules are taken into account in DLS measurements. The hydrodynamic diameter depends on different factors like the electrical conductivity of the solution, intensity (intensity diameter distribution may therefore inherently be weighted to bigger diameter than number distribution, due to the fact that the scattering intensity is proportional to size^6), concentration (multiple scattering causes bigger diameter). The presence of multiple scattering at some concentrations can cause large errors in the measured average hydrodynamic size of the AgNPs. Using the same device, zeta potential was measured. The surface charge is beneficial for determining the stability of colloidal solution (Figure S1, supplementary materials). The average zeta potential value is −18 mV to −34 mV. The high negative potential is associated with high stability, high colloidal quality, and good dispersion of AgNPs as a result of electrostatic repulsion.

### 3.3. Fourier transformed infrared (FTIR) Spectroscopy

**Figure 4:**
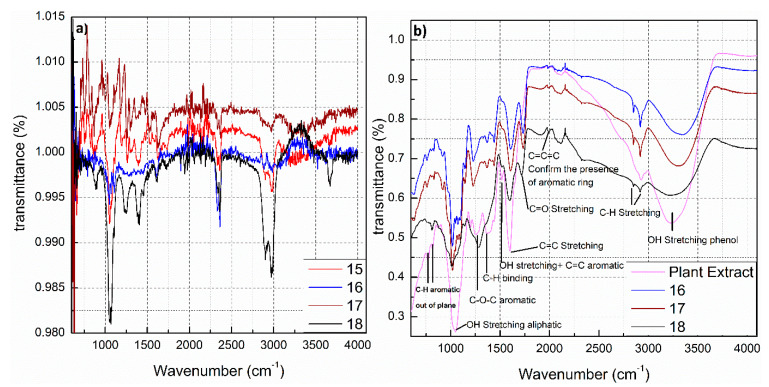
ATR spectra of AgNPs synthesized with St John’s wort extract obtained using a Bruker FTIR Vertex 80 v spectrometer apparatus, (**a**) without vacuum and (**b**) with vacuum.

FTIR spectroscopy was used to prove the existence of an organic compound layer on the surfaces of the nanoparticles and to obtain knowledge of the functional groups on the surface of nanoparticles. AgNPs were examined by thermo-gravimetric analysis (TGA) to prove the existence of organic compounds from *H. Perforatum* extract at the surfaces of silver nanoparticles (Figure S3, supplementary materials). FTIR spectroscopy confirmed the existence of biological components surrounding the AgNPs as a stabilizer. However, due to the complexity of the used plant extract and the fact that most likely more than one substance is adsorbed on the surface of the AgNPs (The detailed composition of the plant extract, the concentration of individual substances, and determination of phytochemicals adsorbed as a protective layer on the surface of AgNPs are currently under investigation and will be included in a follow-up manuscript), interpretation of the FTIR spectra is difficult. The FTIR spectra for AgNPs obtained with *Hypericum perforatum* L. extract are shown in Figure 4. The major peaks of the spectra were assigned to their chemical constituents as summarized in Table S3, supplementary materials. Biomolecules and secondary metabolites originating from the plant extract promote the stability of AgNPs by electrostatic and steric effects. Hydroxyl groups of the biomolecules in St John’s wort, enhance the stability of AgNPs. The hydroxyl-containing biomolecules are protecting the particles for longer periods. Interestingly, the bond vibrations of hydroxyl group (3335 cm^−1^) for AgNPs capped with biomolecules from plant extract lost some of their intensity and had a red shift (to 3338 cm^−1^) compared to the same peak of plant extract alone (Figure 4b). This mainly happened because of the exploitation of hydroxyl groups in the capping and protective action around AgNPs.

### 3.4. Scanning Electron Microscopy (SEM)

**Figure 5:**
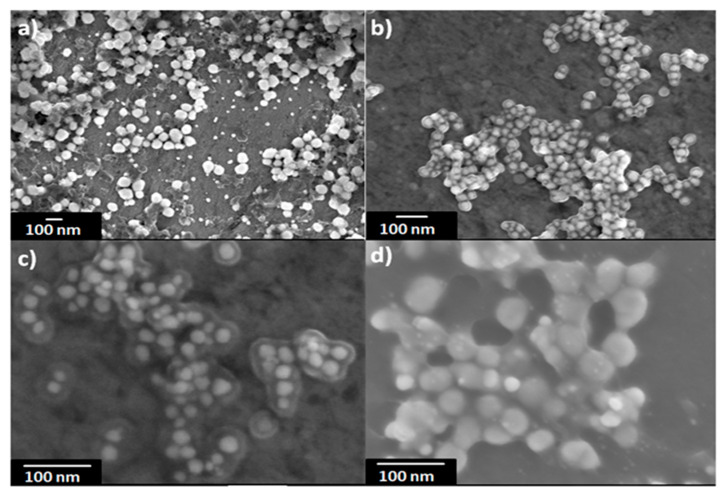
SEM images for the sample 16 at different magnifications, (**a**) 50,000×, (**b**) 100,000×, (**c**) 200,000×, (**d**) 220,000×.

SEM was used to characterize the morphology, size, and size distribution of AgNPs. Samples were prepared by placing the colloids onto a clean polished carbon holder. Afterwards all water was completely evaporated. From Figure 5 it is obvious that the nanoparticles are spherical in shape and exhibit narrow size distribution with a size varying between 20 and 60 nm.

### 3.5. Transmission Electron Microscopy (TEM)

**Figure 6:**
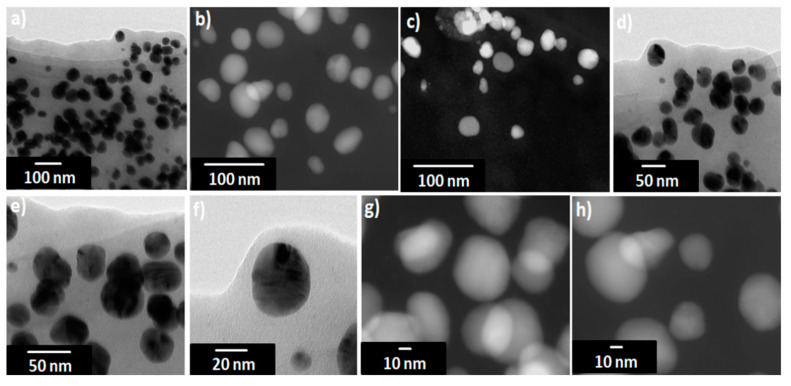
TEM images for the sample 16 at different magnifications. (**a**,**d**) Bright-field transmission electron microscopy (BF-TEM), (**b**,**c**,**g**,**h**) scanning transmission electron microscopy dark-field (STEM-DF) and (**e**,**f**) high resolution transmission electron microscopy (HRTEM).

**Figure 7:**
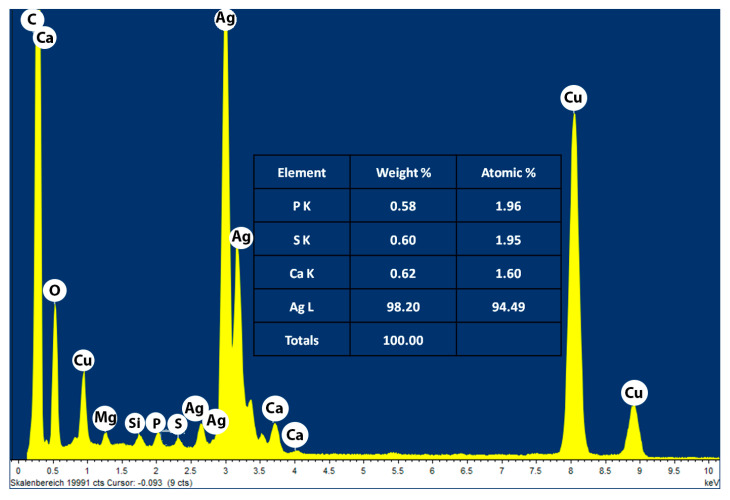
TEM-EDXS analysis of the sample 16 of AgNPs.

TEM images show that the AgNPs are well dispersed and predominantly spherical in shape (Figure 6) with a narrow size distribution which corresponds to the shape of UV-visible spectra. The AgNPs sizes corresponds to that calculated from DLS histogram (Figure 3) with an average diameter of about 20–50 nm. Some agglomerates can be observed, which might be formed during sedimentation within the washing process. The average size estimated was 40 nm for sample 16. In contrast to DLS, the diameter determined by TEM does not include any hydration layer, so with TEM the metal core size is measured. The energy-dispersive X-ray (EDX) spectra were studied from SEM and TEM apparatuses (Figure 7 and Figure S4, supplementary materials). EDX spectra show the presence of silver as an element and strong signals from atoms in the nanoparticles affirm the reduction of silver ions into silver metal. The AgNPs show an optical absorption band peak at 2.8 keV, 3.5 keV, and 3.8 keV corresponding to the binding energies of Ag-L, Ag-M, which are typical for the -absorption of metallic AgNPs. The presence of oxygen and carbon clearly shows that an organic layer is present on the surface of AgNPs. Detected phosphorus originate from the used TEM device and Cu signal is due to high-resolution pole piece and Cu-based TEM grid and does not belong to the specimen. Also, we have identified the amount of oxygen in two different positions (one with AgNPs, one without AgNPs); the percent of oxygen was same, which shows that prepared AgNPs consist of silver and do not contain silver oxides; (see Figures S5 and S6 in the supplementary materials). 

### 3.6. High Resolution Transmission Electron Microscopy and Nano-Diffraction Patterns

**Figure 8:**
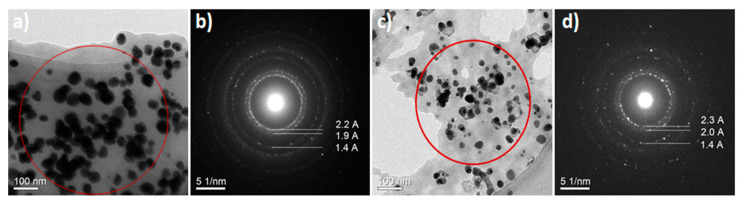
(**b**,**d**) Selected area for electron diffraction (SAED) patterns showing rings corresponding to the crystal planes of AgNPs structure. (**a**,**c**) Bright-field transmission electron microscopy (BF-TEM). The SAED patterns recorded at nominal camera length 1000 mm.

**Figure 9:**
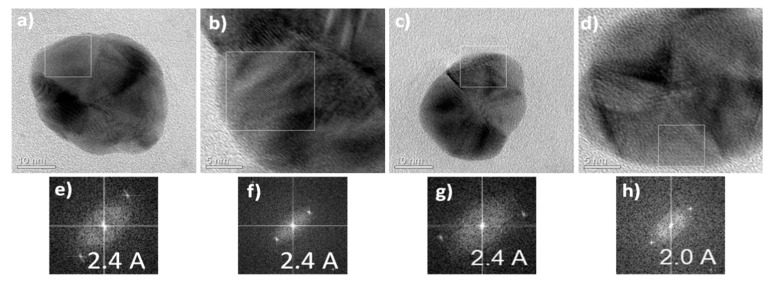
(**a**–**d**) High resolution transmission electron microscopy (HRTEM) images in different orientations of AgNPs from sample 16 and (**e**–**h**) the corresponding characteristic fast Fourier transformation FFTs. this figure shows clearly the crystalline nature of AgNPs through the fully isolated solo lattice fringes (the spacing of 2.4 Å is compatible with the lattice spacing of plane (1 1 1) of pure silver and spacing of 2 Å is compatible with the lattice spacing of plane (2 0 0) of pure silver).

Selected area electron diffraction patterns (SAED) recorded on the regions Figure 8a,c are presented in Figure 8b,d. The observed Debye-Scherer rings are completely enclosed, indicating the Ag nanostructure is highly crystalline in nature (see more explanation about SAED in supplementary materials). The rings change from continuous to dotted as the size of the polycrystalline grains increases. When the size of the grains is too small or the material is completely amorphous, the concentric rings disappears and leaves a halo just around the bright spot in the center, which shows that the electrons are diffracted randomly from the material of the amorphous structure. If the grains in the sample are oriented in a preferred direction, the SAED pattern shows that many rings are partial. If the sample is amorphous, diffuse rings will be obtained, while crystalline samples will result in bright spots, and if the sample is polynanocrystalline (small spots making up a rings, each spot arising from Bragg reflection from an individual crystallite) [95,96,97,98,99,100]. We calculated the d-values (the spacing between lattice planes) and by comparing this value with d-value of different phases of silver in literature, we can identify the type of crystal lattice. For the first three rings (from inside to outside of the central ring) in the SAED images (Figure 8b,d) these values correspond to (111), (200), (220) planes of face-centered cubic (fcc) structure of elemental silver [10]. Figure 9 shows the HRTEM images for sample 16 and the corresponding Fourier spectra (equivalent to optical diffraction patterns), that were obtained by fast Fourier transformation (FFT) process for HRTEM images.

FFTs were used for better understanding of the nanoparticles orientation in relation to an electron beam. These orientations are suggested in the insets. Figure 9 clearly indicates the crystalline nature of AgNPs by very well separated individual lattice fringes (the spacing of 2.4 and 2.5 Å corresponds to the lattice spacing of (111) plane of pure silver and the spacing of 2.0 Å corresponds to the lattice spacing of (200) plane of pure silver). It must be noted that the planes (111) were a little distorted where its value is 2.4 Å compared with bulk value 2.35911 Å as exhibited in FFT pictures for these nanoparticle. Therefore, there is a distortion in the particle of about 2% and 6% (expansion) along the directions [101].

### 3.7. X-ray Diffraction (XRD)

**Figure 10:**
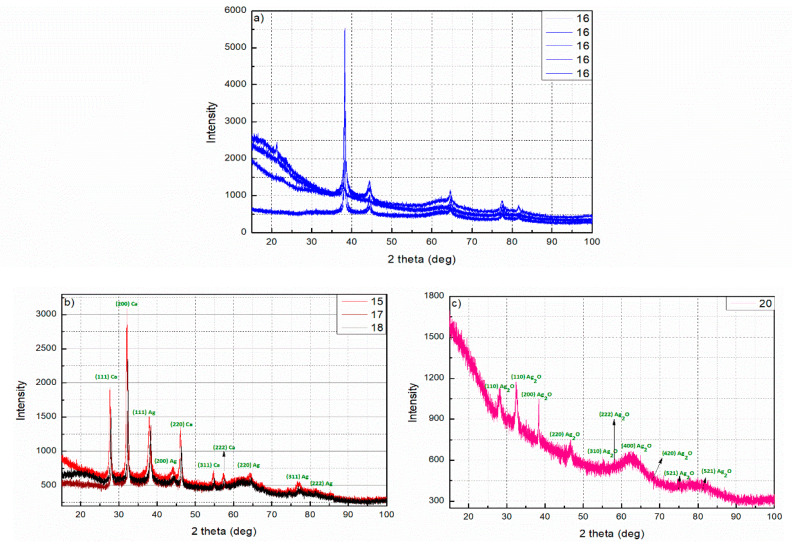
XRD diffractograms for different samples, (**a**) for sample 16 at different periods, (**b**) for samples 15, 17 and 18 which clearly show the formation of calcium with silver in the solution, (**c**) for sample 20 which clearly show the formation of silver oxide.

The X-ray diffraction pattern of AgNPs synthesized by using *Hypericum perforatum*.L as a reducing and capping agent is shown in Figure 10. Indexing process of powder diffraction pattern was carried out and Miller indices (*h k l*) for each peak were determined (Table 2). A number of strong Bragg reflections were observed at 2θ of 38.2461°, 44.4345°, 64.596°, 77.5785°, and 81.7060° (for sample 16 at different times; when repeating the experiment always with the same conditions). These diffraction peaks correspond to the (111), (200), (220), (311), and (222) planes of AgNPs, and confirm the face-centered cubic (fcc) lattice structure of the biosynthesized AgNPs (JCPDS: File No. 03-065-2871). We did not find any unexpected diffractions and this proves that there are no crystalline impurities [102]. All the reflections off the sample 16 agree with fcc structure of pure silver. In materials mono-atomic fcc structure, the strongest reflection should be from (111) plane. This is clearly observed in our sample No. 16. So we can conclude that the degree of crystallization and purity of AgNPs is very high. The diffraction peaks are broad indicating that the size of the crystals is small [103]. The lattice parameter or unit cell edge (a) is 4.07 Å (JCPDS file no.04-0783) for materials which have fcc crystal structure. Using the equation [104]:

$$a=d\;\times \;\sqrt{h^{2}+k^{2}+l^{2}}$$

We calculated the experimental lattice constant from the peaks (111), (200), (220), (311) and (222) in the XRD pattern and it was 4.07 Å (Table 2), which means that that both, theoretical and experimental values are identical.

Here it should be noted that the ratio between the intensities of the (200) and (111) diffraction peaks is slightly less than the theoretical value (Table 3). While this value for plane (220) ranges from smaller to larger than conventional value and it is also for the two planes (311) and (222). This can be attributed to the effect of “preferred orientation.” Besides the signature “broadening” due to the nano-size of the particles, we should also see the contribution due to preferred orientation.

Determination the particles size of the AgNPs for many samples from Debye-Scherrer’s Equation are calculated (Table S4, supplementary materials). The calculated crystallite size of as-prepared Ag-NPs (~24–27 nm) for sample 16 is compatible with the values obtained from SEM and TEM. The crystallite size of Ag-NPs for sample 16 (~10–15 nm) is rather small compared to the average nanoparticle size estimated from SEM and TEM analysis (~25–40 nm), suggesting that there are small nanocrystals in the multiple twined AgNPs. As a result of the crude nature of St. John’s wort extract, it may also contain ions, salts, and metabolites. These ions were reduced during the reaction to get other metals like calcium (Ca) (Figure 10b). In other cases, silver ions may oxidize in the medium of the reaction to obtain silver oxide as is the case in the sample 20 (Figure 10c).

### 3.8. Possible Mechanism of Biosynthesis of Silver Nanoparticles by Plant

In the past years, researchers focused on the synthesis of nanoparticles by green methods, because these methods have a lot of advantages which have been mentioned earlier. One of the most commonly used green methods is using plant extracts. The important question in this case is: Which molecules or substances in the plant extract play a role as a reducing and protective agent? Many compounds (secondary metabolites or photochemicals) were identified, which effectively reduce silver ions and stabilize silver nanoparticles: polyphenols (flavonoids, catechin, phenylpropanes, phenolic acids, anthocyanins, proanthocyanidin, taxifolin, flavones and biflavones), alcoholic compounds, organic acids (tartaric, ascorbic, protocatechuic, oxalic, and malic acid), mono and polysaccharides, amino acids, terpenoids, quinones, an tioxidants, glutathiones, and alkaloids (see Table S5, supplementary materials).

**Figure 11:**
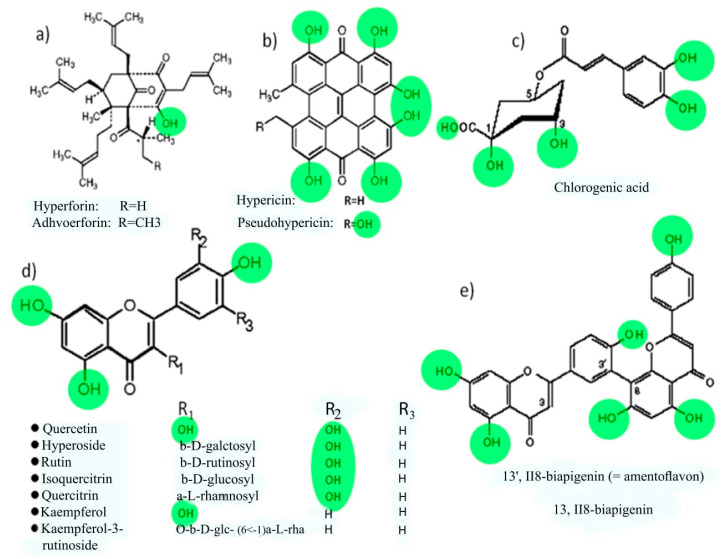
The most important constituents of *Hypericum perforatum* L. (**a**) represent general formula of Hyperforins, (**b**) Hypericins, (**c**) chlorogenic acid, (**d**) Flavonoids and (**e**) Biflavones

As seen in Figure 11 and Table S5 (supplementary materials), polyphenols and flavonoids are the most common compounds which have been revealed to be involved in the green synthesis of AgNPs. The extract of aerial parts from *Hypericum perforatum* L. is rich in polyphenolic compounds (Figure 11) and contains six major bioactive natural products groups: (1) naphthodianthrones (such as hypericin and pseudohypericin), (2) phloroglucinols (such as hyperforin and adhyperforin), (3) flavonoids (such as quercetin, rutin, quercitrin and kaempferol), (4) biflavones (such as 3,8′-biapigenin and amentoflavone), (5) phenylpropanes (such as chlorogenic acid and caffeic acid), and (6) proanthocyanidins (such as dimeric procyanidin B2 and trimeric procyanidin). In addition, there are some amounts of tannins (such as proanthocyanidins), xanthones (such as 1,3,6,7-tetrahydroxyxanthone and kielcorin), essential oils (hydrocarbons and long chain alcohols), and amino acids (such as γ-aminobutyric acid) (Figure 11) [77,105,106].

**Figure 12:**
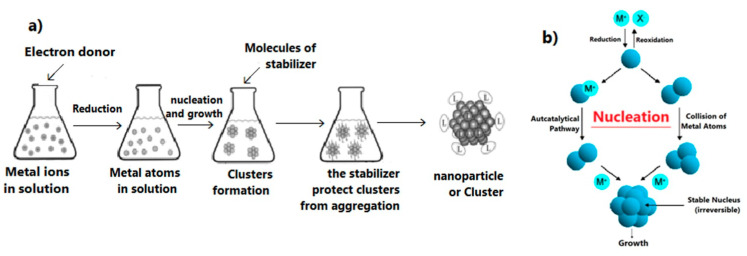
Simplified scheme shows (**a**) the stages of the formation of nanoparticles that are stable and protected from aggregation, (**b**) formation of small clusters and their growth (nucleation and growth).

Currently there is no literature available describing the mechanism for the metabolites reduction and stabilization of AgNPs. The biomolecules existing in the *Hypericum perforatum* L. extract (such as flavonoids, hypericins, hyperforins, mono- and polysaccharides, phenolic acids, etc.,) interact with silver ions and convert them into nanoparticles. The mechanism of AgNPs formation consists of mainly three steps: reduction of ions to get metal atoms, clustering of some atoms resulting in small clusters (Figure 12b), and growing of these clusters and protection of formed NPs by capping agent to prevent aggregation (Figure 12). All of these steps depend on precursor concentration (AgNO_3_), the concentration of reducing and stabilizing agent in the plant extract, pH of medium, temperature and stirring speed [107].

**Figure 13:**
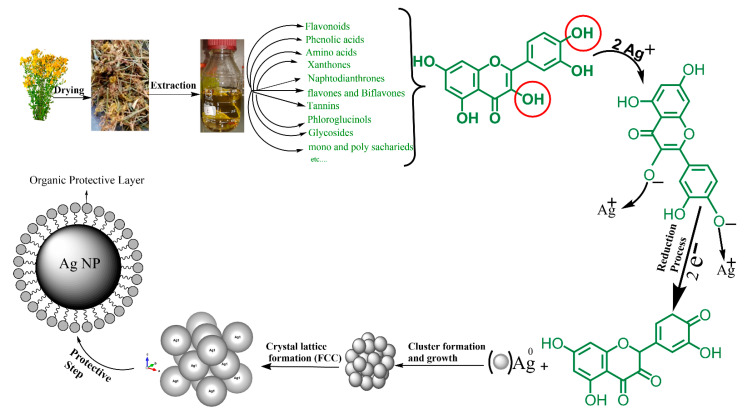
Scheme represents the proposed mechanism of green synthesis of AgNPs from *Hypericum perforatum* L. (quercetin was used as an example, because many of the phytochemicals or secondary metabolites present in this plant extract have a chemical structure similar to the chemical structure of quercetin in terms of the presence of hydroxyl groups associated with aromatic rings). These organic compounds reduce silver ions to silver metal, and when small clusters form and grow, the organic compounds are placed on the lattice planes to force them to grow in specific directions in order to obtain a certain crystal lattice (face centered cubic fcc in our case). At the same time, they prevent the aggregation of the nanoparticles and promote the production of smaller NPs.

The -OH groups present in polyphenols compounds in *H. perforatum* L. such as flavonoids may be responsible for the reduction of silver ions to AgNPs [108]. It is possible that the tautomeric transformation of these compounds from enol to keto form releases a reactive free electron which reduces silver ions to metallic silver [109]. Our data shows that molecules from *H. perforatum* L. extract were responsible for the stabilization of the AgNPs, as FTIR spectra of both, AgNPs and *H. perforatum* L. extract, demonstrated the presence of similar functional groups. Stabilizing biomolecules often contain more than one potential bidentate binding sites that can produce protons such as quercetin: α-hydroxy-carbonyl, β-hydroxy-carbonyl, and catechol having two hydroxyl groups in ortho positions, can produce two protons which can react with nitrate anions, i.e., one molecule of quercetin reduces two silver ions [110,111]. *H. perforatum* L. contains a large amount of polyphenols (phytochemicals or secondary metabolites) which have hydroxyl and ketonic groups. These compounds that have an hydroxyl group linked to carbon atoms in the aromatic ring react with silver ions as acid and reduce Ag ions to Ag metal in the nano size and protect it from aggregation (i.e., the crystals of Ag start to appear and grow from metallic Ag after the supersaturation of hydroxyl complexes Ag-OH) [112,113]. The process continues until the specific growth of all the silver crystal planes, i.e., the stabilizer from the extract will inhibit the growth of high-energy atomic planes. These AgNPs are found in high surface-energy state and tend to turn into their low-surface energy form by aggregating with each other, so the biomolecules from *H. perforatum* are attracted toward the higher-energy crystallite planes and function as protective agent to hamper the growth. Thus, the existence of higher concentrations of reducing and protective agents inhibits the aggregation of nanoparticles and results in smaller size of AgNPs. Figure 13 showed the proposed mechanism of biosynthesis of AgNPs by using *H. perforatum* L. as a reducing and protective agent. Quercetin was used as an example because many of the phytochemicals present in this plant extract have a chemical structure similar in terms of the presence of hydroxyl groups associated with aromatic rings. The phytochemicals that act as a reducing agent are not necessarily the same which play a role as protective agents.

### 3.9. Determination of Antioxidant Activity

Several abiotic stresses lead to the overproduction of highly toxic and reactive oxygen species (ROS). These reactive oxygen species are known to cause damage to DNA, carbohydrates, proteins and fats, build oxidative stress and lead to the induction of various diseases. Nanomaterials and especially AgNPs represent promising candidates in the search for enhanced antioxidants. It has been shown that silver nanoparticles have the capacity to reduce oxidative stress due to their effective redox-active radical-scavenging properties. Moreover, the used plant extract contains bioactive phytochemicals like polyphenols. These bioactive components could display potential antioxidant activity. Since the silver nanoparticles presented here are synthesized using plant extracts, this can result in enhanced antioxidant activity and thus open exciting possibilities for the development of superior antioxidants. Antioxidant testing methods are different and it is difficult to compare one method to another. Therefore the total capacity of the antioxidant cannot be assessed on the basis of a single antioxidant test form, so use of combination of various tests is always useful.

#### 3.9.1. 1-1,1-Diphenyl-2-picryl Hydrazyl Assay

**Figure 14:**
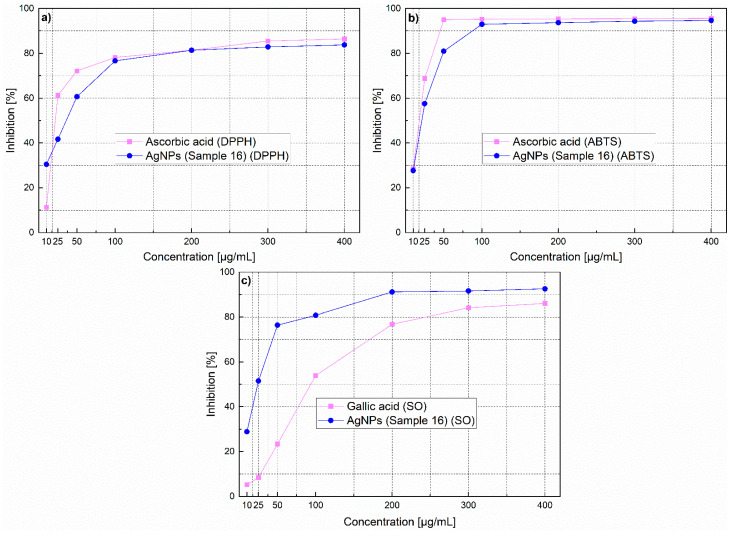
Antioxidant activity of AgNPs (**a**) DPPH free radical scavenging activity, (**b**) ABTS radical scavenging activity and (**c**) superoxide anion radical scavenging activity.

The antioxidant activity of AgNPs was measured with regard to their scavenging activity of the stable 1, 1-diphenyl-2-picryl hydrazyl (DPPH) free radical. DPPH is commonly used to detect the scavenging capacity against radicals in the chemical assay. DPPH is a renowned radical and a snare to other radicals (scavenger). The inhibitory concentration 50% (IC_50_) was determined from calibration curves, obtained from different concentrations of the different AgNPs. In the present study, AgNPs showed potential free-radical scavenging activity, this means that AgNPs were able to reduce the stable, purple-colored radical, DPPH into the yellow-colored DPPH. In Figure 14a, the IC_50_ values are presented. AgNPs showed an IC_50_ value of 35.88 μg/mL which was near that of standard ascorbic acid (IC_50_ = 35.44 μg/mL) (Figure 14a). This demonstrates higher effectiveness of investigated AgNPs when compared to others described in the literature [114,115,116,117,118]. AgNPs exhibited scavenging average ranging from 30.47% to 76.63%, at concentrations 10–100 µg/mL. This indicates that the resulting activity of the AgNPs is not only attributed to the nanosize, but it is further enhanced by the protective agents (chemical constituents originating from the extract). The protective layer at the surface of AgNPs may include acid, sugars, hydroxyl-phenolic and phenolic compounds, flavonoids and others, which may be an additional source of the antioxidant activity.

#### 3.9.2. 2-2,2′-Azino-bis-(3-ethylbenzothiazoline-6-sulfonic Acid Radical Cation Scavenging Assay (ABTS)

ABTS antioxidant activity was measured as described by S. Chanda et al. [119] with some minor modifications, as described in the methods section. An inhibition of 27.7–92.6% was obtained in the concentration range of 10–100 µg/mL (Figure 14b). By increasing the concentration of AgNPs, the ABTS cation radical scavenging activity significantly increases, while at concentrations above 100 µg/mL no further increase could be observed. The IC_50_ value of AgNPs was 26.78 µg/mL, which was near that of standard ascorbic acid (IC_50_ = 23 μg/mL). This indicates that AgNPs prepared with our method inhibit cation radicals more effectively than AgNPs prepared by other biological methods [115,117,120,121].

#### 3.9.3. Super Oxide Anion Radical Scavenging Assay (SO Assay)

Superoxide anions are extremely grave radicals and if not put down will cause the formation of other risky radicals such hydrogen peroxide (H_2_O_2_), peroxyl (ROO˙), hydroxyl (OH˙), superoxide (O_2_˙), singlet oxygen (O_2_), nitric oxide (NO˙), peroxinitrite (˙ONOO), and cyanide (CN˙). Superoxide causes the generation of vigorous and dangerous hydroxyl radicals and singlet oxygen, which both contribute to oxidative stress. Results of the superoxide anion radical scavenging activity of AgNPs and gallic acid are given in Figure 14c. As shown in the figure, the quenching of super oxide radicals increase with increasing the concentration of AgNPs. AgNPs at concentrations 10–400 µg/mL showed scavenging ranging from 29 to 93% (Figure 14c). The IC_50_ value of AgNPs was 27.77 µg/mL while that of gallic acid (the standard in this assay) was 94 µg/mL indicating that the superoxide anions scavenging ability of AgNPs is much better than that of gallic acid. Superoxide radical scavenging activity of investigated AgNPs was higher as described in the literature for AgNPs synthesized by other methods [115,117,120,122,123,124,125,126,127].

### 3.10. Determination of Time- and Dose-Dependent Cytotoxicity of Biosynthesized AgNPs

**Figure 15:**
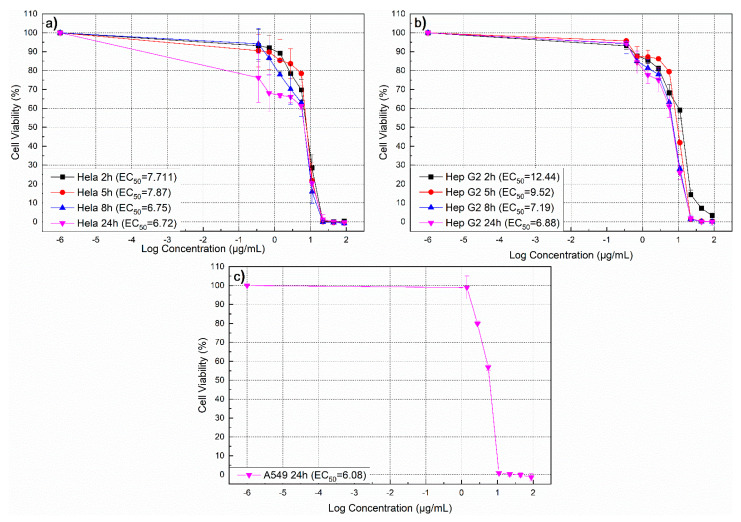
Relative cell viability (%) of (**a**) Hela, (**b**) Hep G2 and (**c**) A549 cells as a function of AgNPs concentration for 2, 5, 8, and 24 h determined by cell titer-blue cytotoxicity assay. The experiments were performed with four replicates and standard deviation was calculated.

In CTB test for evaluation of the cell viability, viable cells turn blue resazurin into pink resorufin generating a fluorescent signal, while nonviable cells cannot do that because they lost their metabolic activity. Both, the influence of exposure time, and AgNPs dose were studied. The time ranged between 2, 5, 8 and 24 h, and the dose range of AgNPs synthesized utilizing St John’s wort extract was between 2.5 μg and 88 μg/mL. The range of viability observed for HeLa cells, Hep G2 cells, and A549 cells are shown in Figure 15a–c respectively. Actually and as evident from Figure 15, by increasing the concentration of AgNPs, cell viability of the three cell types studied in this research significantly decreased. For Hela cells, no difference in cell viability was observed for different exposure times (2, 5, 8, and 24 h), but for the HepG2 cells, the effect depends on the exposure time.

A549 cells were studied only after 24 h exposure to AgNPs. Distinctly, the cell viability significantly dropped by about more than 90% at low AgNPs concentrations in a range of 11−20 µg/mL. AgNPs concentration range between 11 and 20 µg/mL has significant effect on cell viability. AgNPs affected cell viability for each of the three studied cancer cell lines dependent on exposure time and concentrations. No significant effect of the exposure time was detected for HeLa cells (IC_50_ value after 2 h incubation was close to the value after 24 h incubation) compared with HepG2 cells (IC_50_ value at 2 h incubation was about twice the value after 24 h incubation). Cell viability of three cell lines (HeLa, HepG2, and A549 cells) decreased significantly after incubation with AgNPs at various concentrations, and toxic effects of AgNPs on HeLa cells after 2 h (IC_50_ = 7.711) was more pronounced when compared to that on Hep G2 cells (IC_50_ = 12.44), while after 24 incubation hours, the values were closer to the three cancer cells studied (IC_50_ = 6.72 for Hela cells, IC_50_ = 6.88 for Hep G2 cells, and IC_50_ = 6.08 for A549 cells). These results proved the high cytotoxic effect of AgNPs on HeLa, HepG2, and A549 cells compared with previous research [60,128,129,130,131,132,133,134,135] respectively, and it can be assumed that this higher cytotoxicity originates from phenolic compounds from St John’s extract that coat these nanoparticles. To achieve targeting we are currently working on the functionalization of the AgNPs with cancer-specific aptamers. Thus our goal is to develop nanoparticles that have a size that allows entering to the tumor site via EPR effect and to the cancer cells via aptamers-mediated endocytosis, and are toxic to the cancer cell.

## 4. Conclusions

A simple one-pot green synthesis of stable AgNPs using aqueous St John’s wort extract was reported. Preparation was found to be efficient in terms of stability as well as reaction time and excluded additional stabilizers/reducing agents. It proves to be an environmentally friendly, rapid green approach for the synthesis and provides a cost effective and an efficient way to synthesize AgNPs. Thereby, this reaction path satisfies all the requirements of a green chemical process. The synthesized nanoparticles were monodisperse, spherical, 20–50 nm in size, crystalline in nature, and showed absorption at 392–420 nm. XRD study showed the face-centered cubic lattice of AgNPs, EDXS analysis gives the optical absorption peak approximately at 2.8 keV, 3.5 keV, and 3.8 keV, confirming the formation of metallic silver. TEM and SEM analysis showed that most of the particles were spherical in shape with size 20–35 nm. Prepared particles were surrounded with natural compounds from *Hypericum perforatum*. The particles are stabilized by a protective layer composed of molecules originating from the plant extracts which contain hydrophilic functional groups. The synthesized AgNPs showed very high antioxidant activity against 2, 2′-azino-bis-(3-ethylbenzothiazoline-6-sulfonic acid) radical cation, super oxide anion radical, and 2,2-diphenyl-1-picrylhydrazyl radical, at very low concentrations. Moreover, the AgNPs decreased cell viabilities of Hela, Hep G2, and A549 cells within a short time period of 2 h. AgNPs-toxicity toward cells may be related to various properties such as high surface area, surface charges and capping layer components, agglomeration, and lipophilicity. Organic molecules from St John’s Wort coating AgNPs, which produce high surface charges, may play an additional role in AgNPs-toxicity. In subsequent studies we will use cancer cell-specific aptamers to achieve selective targeting of cancer cells. These biological synthesized and aptamer-functionalized AgNPs could be useful in the medical field for the treatment of cancer. AgNPs can not only be used as drug carriers but also represent the drug itself. In this case high toxicity of the AgNPs is a favorable feature. To achieve targeting we are currently working on the functionalization of the AgNPs with cancer-specific aptamers. Thus our goal is to develop nanoparticles that have a size that allows entering to the tumor site via EPR effect, to the cancer cells via aptamers-mediated endocytosis and are toxic to the cancer cell. This will be further investigated in our future work on Allah’s will.

## Figures and Tables

**Table 1:** Protocols used for AgNPs preparation, more details can be found in the supplementary materials.

Sample Number	Volume of Silver in the Sample [mL] (Concentration: 0.001 M)	Volume of Plant Extract in the Sample [mL]	Other Conditions
1	10	0.6	The sample was diluted up to 10% [23]
2	5	10	Mixture was heated to 37 °C while stirring at 200 rpm for 72 h [89]
3	95	5	-
5	10	10	[27]
6	9	1	Sample was stirred at 200 rpm [26,30] for 48 h without heating
7	5	1	Sample was put it in a microwave oven for 5 to 15 min [90]
8	5	1	Sample was placed in the incubator (37 °C) for one hour [90]
10	50	2	[32]
11	20	200	Sample was heated in a water bath at a temperature of 75 °C for one hour
12	5	30	Room temperature for 2 days
13	5	95	Sample was heated to 65 °C while stirring at 200 rpm for 6 h
14	15	1	Sample was heated to 37 °C while stirring at 200 rpm for 2 h
15	5	5	Sample was heated to 37 °C while stirring at 400 rpm for 2 weeks [31]
16	5	15	Sample was heated to 60 °C while stirring at 700 rpm for 4–6 h
17	5	25	Sample was heated to 37 °C while stirring at 400 rpm for 2 weeks
18	5	30	Sample was heated to 37 °C while stirring at 400 rpm for 2 weeks
19	5 mL (Concentration: 0.01 M)	15	Sample was heated to 60 °C while stirring at 700 rpm for 4 h
20	5 mL (Concentration: 0.1 M)	15	Sample was heated to 60 °C while stirring at 700 rpm for 2 h

**Table 2:** Calculation of Miller indices and lattice constant for sample 16 from diffraction angles.

2θ (o)	Sin 2θ	Sin 2θ/Sin 2θ for the First Peak	(Sin 2θ/Sin 2θ for the First Peak) × 3	(*h*^2^ + *k*^2^ + *l*^2^)	(*hkl*)	Sin 2θ/ *h*^2^ + *k*^2^ + *l*^2^	d (Å)	a (Å)
38.2461	0.1073	1	3	3	111	0.036	2.3531	4.07
44.4345	0.14297	1.332	3.996	4	200	0.036	2.0387	4.07
64.596	0.28551	2.661	7.983	8	220	0.036	1.4427	4.07
77.5785	0.39245	3.658	10.974	11	311	0.036	1.2306	4.07
81.7060	0.42787	3.988	11.963	12	222	0.036	1.1785	4.07

**Table 3:** Ratio between the intensities of the diffraction peaks for sample16.

Diffraction Peaks	Sample Value	Theoretical Value
(200) and (111)	from 0.24 to 0.38	0.45
(220) and (111)	from 0.19 to 0.35	0.24
(311) and (111)	From 0.15 to 0.25	0.25

## Data Availability

The data presented in this study are available on request from the corresponding author.

## Supplementary Materials

The following are available online at https://www.mdpi.com/2079-4991/11/2/487/s1. Figure S1: Zeta potential for best samples. Figure S2: Isoelectric point for sample 16 and it is almost 1.18. Figure S3: TGA curve of *Hypericum perforatum* L-stabilized silver nanoparticles (sample 16), showing the loss of weight from organic compounds (secondary metabolites) placed on the surfaces of nanoparticles as a capping agent when the temperature increases. Figure S4: SEM-EDX analysis of the sample 16 of Ag NPs as deposited on FTO glass. Figure S5: TEM-DF images in two different places, (a) without silver nanoparticles (b) with silver. Figure S6: nanoparticles TEM-EDX spectra for the sample 16 in two different places, Spectrum 3 with no silver nanoparticles and Spectrum 4 with silver nanoparticles. Table S1: The data λ_max_ (nm) of absorption peaks for different samples, absorption max, peak width (FWHM), molar extinction coefficient which we obtained from the reference study [1] and the final concentration of various sizes of biosynthesized silver nanoparticles. Table S2: Hydrodynamic diameter for other samples. Table S3: The following table lists infrared spectroscopy absorptions bands by its distinctive frequency regions. Table S4: Calculation of diffraction angle, FWHM and crystallite sizes for sample 16 at different times and some other samples of biosynthesized silver nanoparticles. Table S5: Plant components of plant material extract which act as a capping and reducing agent during green synthesis of AgNPs from different plant species.
