# Supplementary material for: Hypericum perforatum L.-Mediated Green Synthesis of Silver Nanoparticles Exhibiting Antioxidant and Anticancer Activities
Source: Nanomaterials (Basel). 2021 Feb 14;11(2):487. doi: 10.3390/nano11020487 (PMC7918618; doi:10.3390/nano11020487)
Supplement: Supplementary file 1 [file nanomaterials-11-00487-s001.pdf]

nanomaterials

Hypericum perforatum L.-Mediated Green Synthesis of Silver Nanoparticles Exhibiting Antioxidant and Anticancer Activities

Alahmad Abdalrahim [1,*], Armin Feldhoff [2], Nadja C. Bigall [2], Pascal Rusch [2], Thomas Scheper [1] and Johanna-Gabriela Walter [1,*]

[1] Institut für Technische Chemie, Leibniz Universität Hannover, Lower Saxony, Germany; scheper@iftc.uni-hannover.de
[2] Institut für Physikalische Chemie und Elektrochemie, Leibniz Universität Hannover, Lower Saxony, Germany; armin.feldhoff@pci.uni-hannover.de (A.F.); nadja.bigall@pci.uni-hannover.de (N.C.B.); pascal.rusch@pci.uni-hannover.de (P.R.)
* Correspondence: alahmad@iftc.uni-hannover.de (A.A.); walter@iftc.uni-hannover.de (J.-G.W.); Tel.: +49-511-762-2773 (A.A.)

Citation: Abdalrahim, A.; Feldhoff, A.; Bigall, N.C.; Rusch, P.; Scheper, T.; Walter, J.-G. *Hypericum perforatum* L.-Mediated Green Synthesis of Silver Nanoparticles Exhibiting Antioxidant and Anticancer Activities. *Nanomaterials* **2021**, *11*, 487. https://doi.org/10.3390/nano11020487

Received: 25 January 2021
Accepted: 9 February 2021
Published: 14 February 2021

Publisher's Note: MDPI stays neutral with regard to jurisdictional claims in published maps and institutional affiliations.

Copyright: © 2021 by the authors. Licensee MDPI, Basel, Switzerland. This article is an open access article distributed under the terms and conditions of the Creative Commons Attribution (CC BY) license (http://creativecommons.org/licenses/by/4.0/).

Experimental

Biosynthesis of silver nanoparticles:

Measurement of concentration through absorption peak in UV-VIS Spectrum:

Table S1. The data λ_{max} (nm) of absorption peaks for different samples, Absorption $_{max}$, Peak Width (FWHM), molar extinction coefficient which we obtained from the reference study [1] and the final concentration of various sizes of biosynthesized silver nanoparticles.

Sample Number	Absorption Peak maximal λ_{max} (nm)	Absorption $_{max}$	Peak Form	Peak Width (FWHM)	Length of the cuvette (cm)	ϵ $M^{-1}Cm^{-1}$ $X10^{8}$	C X 10^{-10} mol/L
1	440	0.31	Wide and very low	55,3388	0.1	880	0.3523
2	430	0.182	Sharp Peak with shoulders	75.32553	0.1	700	0.26
3	430,5	0.101	Wide peak with shoulders	139.40402	0.1	719	0.139
5	442	0.076	Low and expanded	61.52643	0.1	920	0.0826
6	418,2	0.071	Low wide peak with shoulder	102.4439	0.1	477	0.149
7	415	0.099	Small sharp peak with shoulder	61.00594	0.1	415	0.239
8	445	0.079	Small and wide peak with shoulder	68.19435	0.1	958	0.0793
10	442	0.017	very small peak and extended with shoulder	99.2931	0.1	910	0.0187
11	445	0.053	Wide peak without shoulders	131.46316	0.1	967.6	0.0548
12	427	0.177	High and sharp peak with shoulder	79.72299	0.1	658	0.269
13	428	0.012507	Broad peak very small	123,5337	0.1	678	0.0185
14	415	0.1	Sharp small peak without shoulder	64.44719	0.1	390	0.256

15	460	0.435	Sharp peak without shoulders	151.81393	0.1	1162	0. 387
16	442	0.962	Sharp peak without shoulders	113.5924	0.1	920	1.05
17	460	0.295	Small peak but sharp with shoulder	121.67788	0.1	1162	0.254
18	448	0.453	High and wide peak with shoulders	153.02564	0.1	1000	0.453
19	442	0.162	Wide and small peak without shoulders	125.21569	0.1	920	0.176

DLS (Dynamic Light Scattering):

Table S2. Hydrodynamic diameter for other samples**.**

Sample Number	hydrodynamic diameter [nm]	Sample Number	hydrodynamic diameter [nm]
2	172.3	14	78.91
3	87.71	15	78.05
7	167.71	16	70.72
12	109.1	17	73.20
13	145.1	18	88.16
19	171.072	20	211.048

Isoelectric point

The point of zero charge (Isoelectric Point, IP) is the pH at which the electrical charge density on the surface is zero. Usually it is determined in relation to pH of electrolytes, and the point of zero charge value is assigned to a given colloidal particle (as in our case) or substrate. We aimed to determine the isoelectric point of the AgNPs for three reasons:

First: to know at which pH value will be the electrical charges on the surfaces of silver nanoparticles in the colloidal solution zero. Second: is the stability of nanoparticles in the colloidal solution dependent on electrostatic Repulsion because nanoparticles will fall down immediately in the solution when these forces become zero. Third: if the bond between the protective layer and the surface of the nanoparticles is of the type:

● Non-covalent bonds: which they are classified into various categories like hydrophobic effects, pi effects, electrostatic and van deer Waals forces.

● Chemisorptions: is a kind of adsorption which includes a chemical reaction among the adsorbate and the surface.

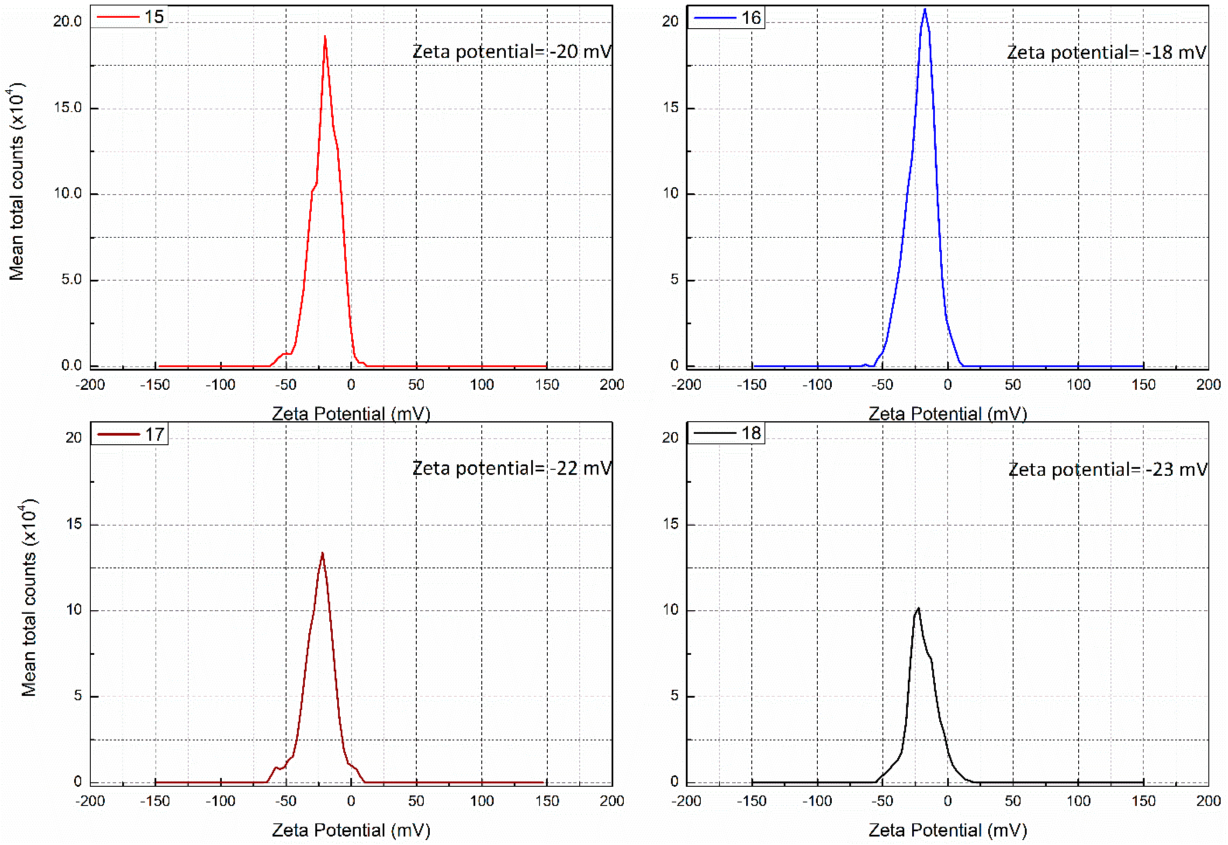

Figure S1. Zeta Potential for best samples

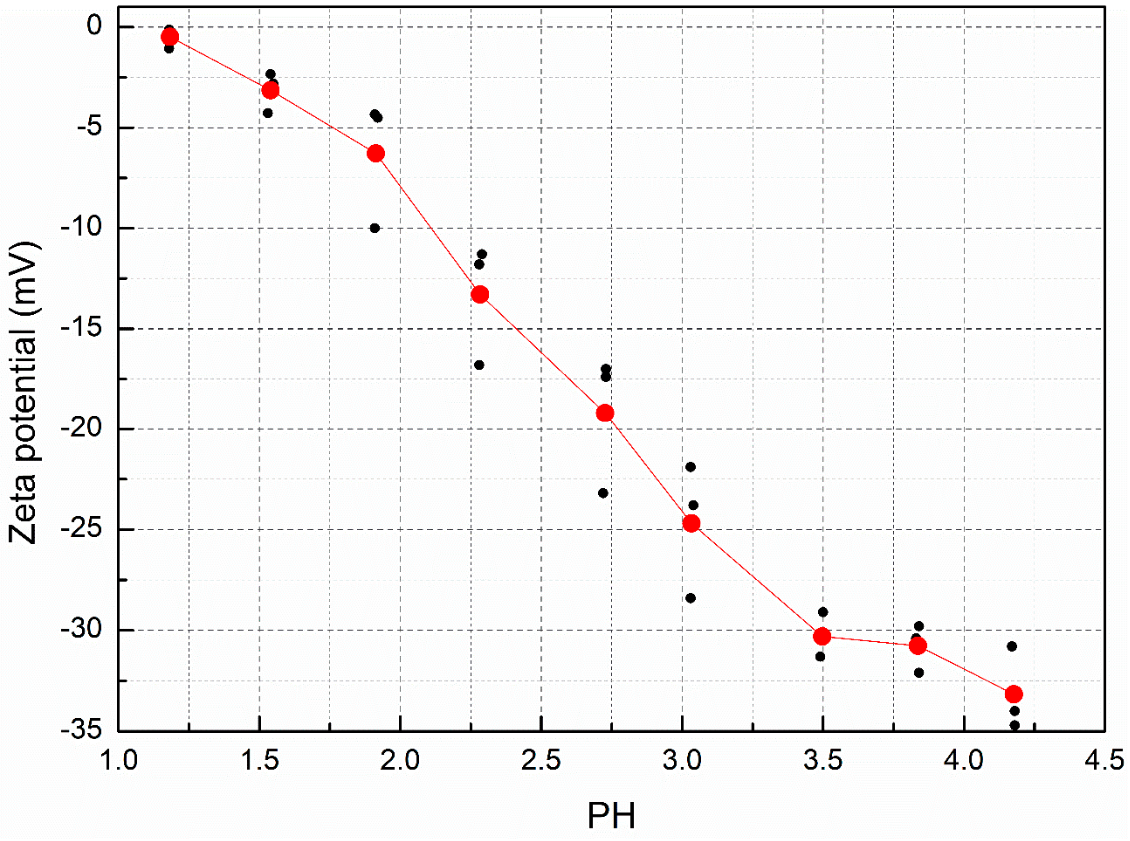

Figure S2. Isoelectric point for sample 16 and it is almost 1.18.

(FTIR) –Spectroscopy:

Table S3. The following table lists infrared spectroscopy absorptions bands by its distinctive frequency regions.

Frequency oder Characteristic Absorptions (cm^{-1})	Functional Group and Type of Vibration[2-5]	Frequency oder Characteristic Absorptions (cm^{-1})	Functional Group and Type of Vibration [2-5]
672.5	C-H aromatic out of plane bending	1527	Stretching OH+ C=C aromatic
763	C-H aromatic out of plane bending ortho	1548	Stretching OH+ C=C aromatic
809	Substituted aromatic ring, C-H aromatic out of plane bending	1617 sometimes1640	Stretching CO+ Stretching CH (flavonoidic structure)
870,880,892	Substituted aromatic ring, C-H aromatic out of plane bending	1733.7	C=O α, β or aryl conjugation
1017	Stretching OH (aliphatic)	1738	Str. C=O Normal
1050	Stretching OH (aliphatic) or S=O sulfoxide	1748	C=O
1066	Stretching OH (aliphatic)	1923, 1934	These peaks confirm the presence of aromatic ring
1077	C-O	1988	These peaks confirm the presence of aromatic ring
1103.6	C-O	2338	C-H bending
1145.5	C-O	2341	C-H bending
1204 sometimes 1236	Stretching OH (aromatic) her said from 1202-1236	2360	
1230, 1242, 1251	C-O-C aromatic	2362	
1265	Stretching OH	2849	Str C-H
1338 sometimes1330	Stretching OH	2917	Str C-H
1374	Stretching OH or C-H bending	2901, 2972, 2987	C-H sp^3 stretch
1394, 1406	OH group of phenolic compound or CH_3	3216	
1443	C-H bending	3385 sometimes 3388	OH stretching in phenols
1451.5	Stretching CH_2+ Stretching OH	3685, 3674	
1473	Str. C=C aromatic	3853.6	
1382	C-H bending	1056	C-O

Thermal gravimetric analysis (TGA):

AgNPs were examined by Thermo-gravimetric analysis (TGA) (TGA/DSC 3+" from the company Mettler-Toledo) to prove the existence of biologically active secondary metabolites from H. Perforatum extract at the surfaces of silver nanoparticles. The progressive increase in temperature was adjusted between 25 °C – 1000 °C at heating rate of 0.5 °C/minute in flowing N_2.

The TGA diagram of the covered silver nanoparticles synthesized using *Hypericum Perforatum* L. extract (Figure S 3) exhibited a stable weight loss in the temperature range of 92-493 °C. The weight loss of the capped-AgNPs was a result of adsorption of bioorganic metabolites at the surface of AgNPs (protective agent) and it was almost between 40-60 % with moisture which is about 9 percent, this depends on the number of washing times with Vivaspin tubes and the conditions of centrifugation used. Figure S 3 shows the progressive degradation of capped-AgNPs in three steps with an increase in temperature. The first phase of degradation which happened between 92 and 164 °C with a weight loss of 6.93% and this weight loss may be attributed to surface adsorbed H_2O molecules and some molecules of hexose ring. The second and third phase of the weight loss occurred between 170-315 and 324-492 °C consecutively, with a weight loss of 31.11 and 20.84 % respectively, this weight loss was due to combustion of the protective layer (the metabolites from St john`s extract which acts as a capping agent at the surface of AgNPs when formed) which decomposition in two steps. In summary the results of TGA analysis correspond to the results of FTIR and prove that resulted silver nanoparticles from synthesis were mixed nature with strongly coordinated metal organic framework.

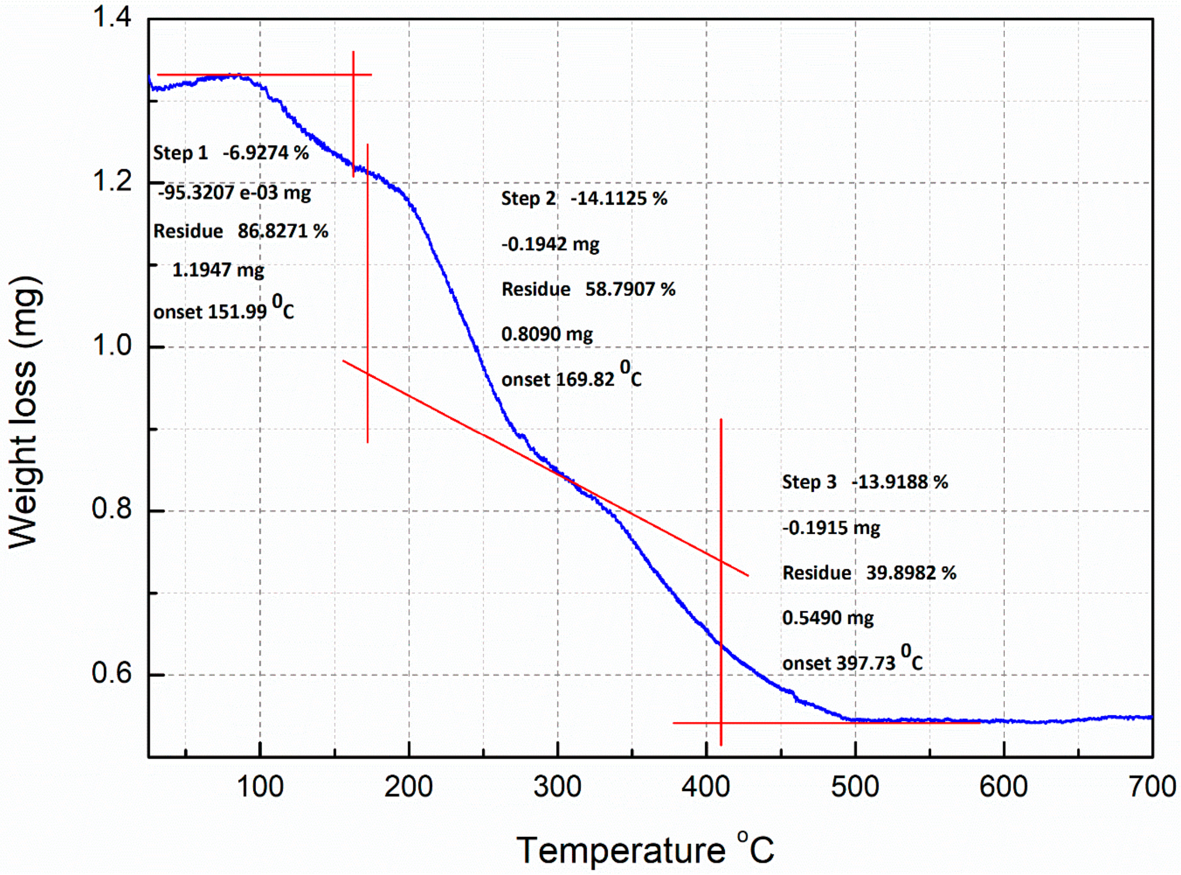

Figure S3. TGA curve of *Hypericum Perforatum* L-stabilized silver nanoparticles (sample 16), showing the loss of weight from organic compounds (secondary metabolites) placed on the surfaces of nanoparticles as a capping agent when the temperature increases.

Scanning electron microscopy (SEM):

Energy dispersive X-ray spectroscopy (EDX): Oxygen and carbon appearance clearly shows that an extracellular organic layer covers the surface of AgNPs. The occurrence of another element in the photomicrograph could be on the grating base FTO glass which is used for the analysis.

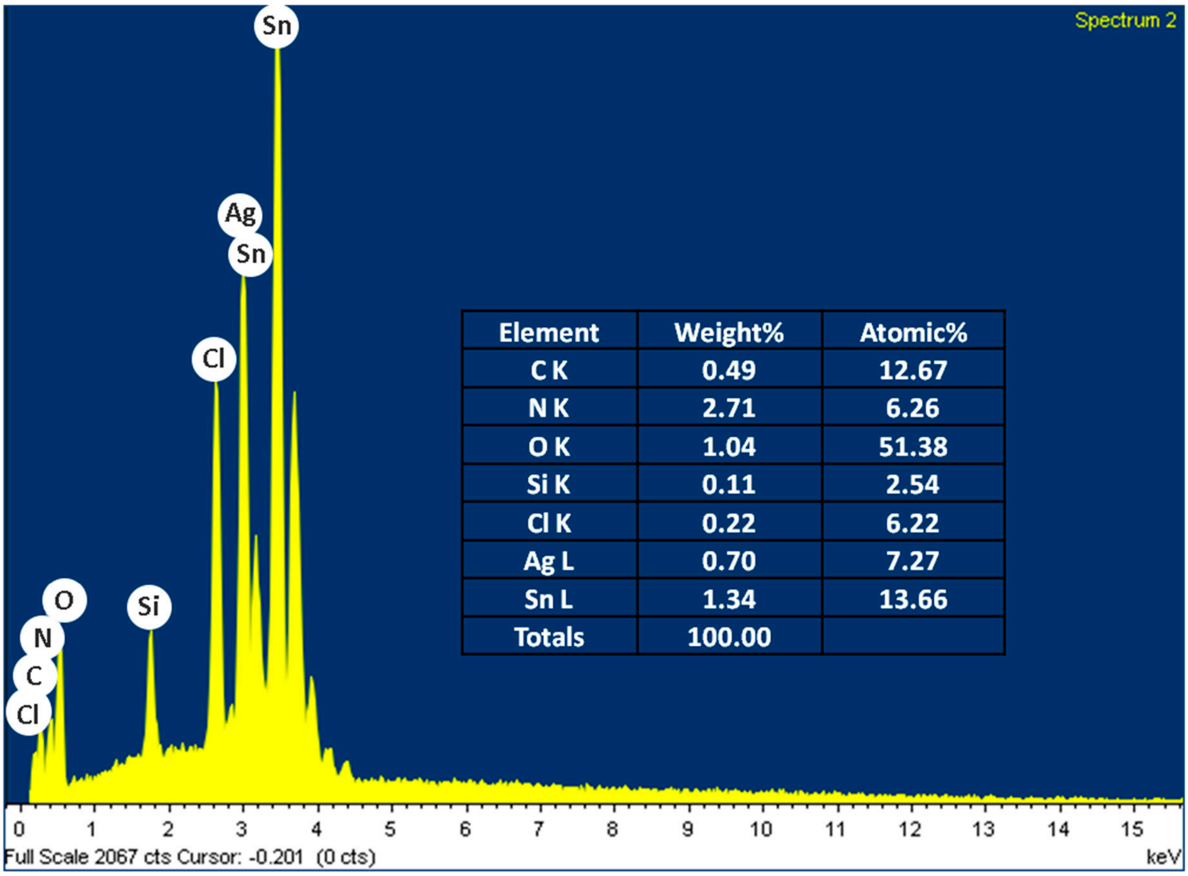

Element	Weight%	Atomic%
C K	0.49	12.67
N K	2.71	6.26
O K	1.04	51.38
Si K	0.11	2.54
Cl K	0.22	6.22
Ag L	0.70	7.27
Sn L	1.34	13.66
Totals	100.00	

Figure S4. SEM-EDX analysis of the sample 16 of Ag NPs as deposited on FTO glass

Transmission electron microscopy (TEM):

Through the two images Figure S5 and the spectrum Figure S6 we note the following: in a place where there is no silver, the concentrations of all the other elements are same, especially oxygen, this proves that what we got is only the silver metal and there are no silver oxides in the samples. We note that the concentration of carbon is less where there is silver metal. This indicates that silver is surrounded by a protection layer from the organic compounds present in the extract, which means, it proves the presence of the organic layer on the surfaces of nanoparticles.

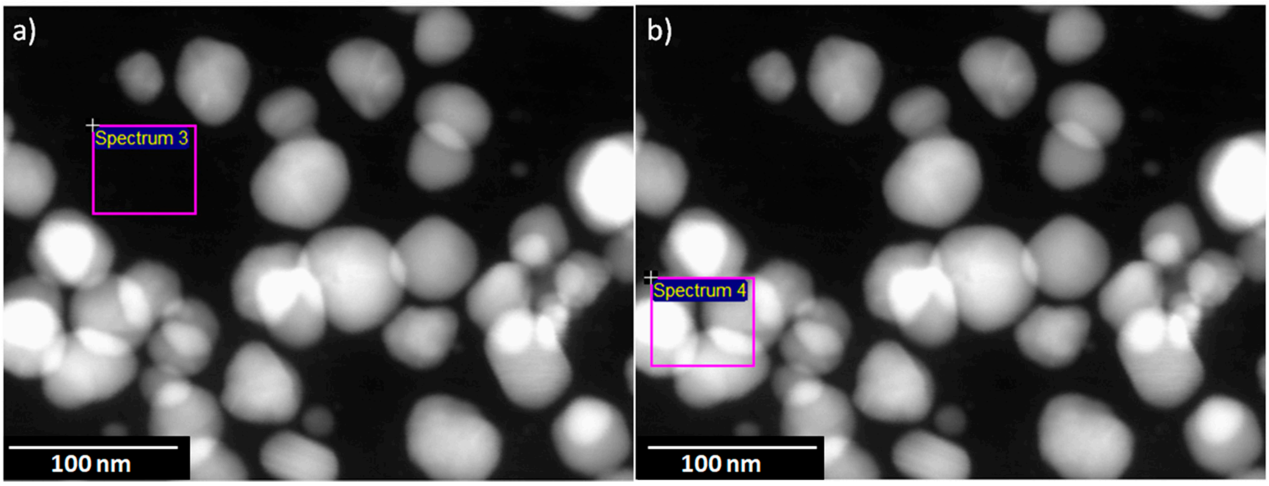

Figure S5. TEM - DF images in two different places, a) without silver nanoparticles b) with silver nanoparticles.

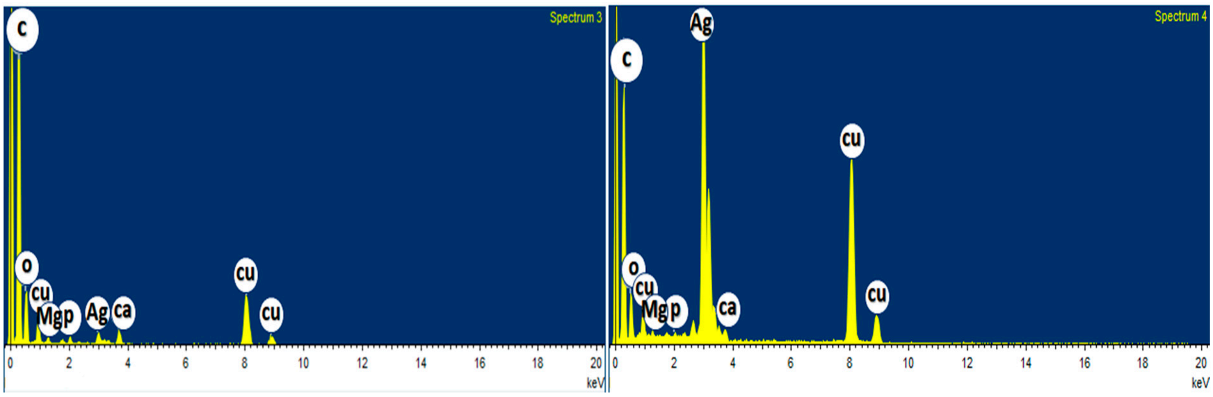

Figure S6. TEM-EDX spectra for the sample 16 in two different places, Spectrum 3 with no silver nanoparticles and Spectrum 4 with silver nanoparticles.

High resolution transmission electron microscopy and nano-diffraction patterns [6-11]:

SAED is a qualitative analysis method of crystal structures from a spot diffraction pattern, which is obtained from illumination of a parallel electron beam on a specimen. When entering a selector (chosen-region) slot into the image level of the objective lens, is obtained a deviation pattern from a sample area of a various 100 nm diameter. This method enables us to identify the lattice type, lattice parameters and crystallographic orientation of this selected area. To analyze patterns of SAED, we integrate the geometric relationship and Bragg's equation in the reciprocal space:

$$2d \sin \Theta = \lambda \qquad \text{(Bragg's law)}$$

$$\text{Tan } 2\theta = D/L \qquad \text{(geometric relationship in reciprocal space)}$$

Where λ is the wavelength of the electron beam, d is spacing between planes, θ is the diffraction angle, D is the distance between rings on the SAED pattern, L is camera length of TEM apparatus. For too small diffraction angle θ, tan2θ equals to 2sinθ, thus for the spots on the SAED pattern; d-spacings can be calculated by:

$$d = D/\ (\lambda\ L)$$

Each ring or spot in SAED pattern corresponds to a lattice plane of a specific miller index in single crystal or a group of lattice planes of the same miller index family in the

polycrystalline sample. Whole spots on the pattern could be indexed. If the electron beam is fixed and the sample rotates, several ring/spot will be activated, and another ring/spot might die far. It depends on the diffraction situations described over. The diffraction pattern can be considered as a finger print for a certain crystal. Generally, the electron beam diffracted by a single crystal produces a diffraction spots pattern, but the specimen which consist of big number of little randomly distributed grains produce a continued rings. This is because all these grains contribute to the formation of diffraction pattern. Radius of the spots is inversely proportional to the interplanar spacings d_{hkl} of lattice planes of crystals.

The method to index the SAED pattern can be explained step by step:

(1) The diameter (2R), of each ring is measured using some image processing software such as the image tool.

(2) The radius value (R) of the diameter (2R) is taken.

(3) By comparing this value with d-value of different phases of silver in literature (Joint Committee on Powder Diffraction Standards (JCPDS) or ICSD data base), we can identify or know the type of crystal lattice.

X-ray Diffraction (XRD):

The material consists of crystals in which the atoms are organized in a specific order. Since the wavelength of the X ray is close to the distance between atoms in these crystals, the X-ray diffraction suffers if they are coated on the material. The detector records the angles that are curving at the crystalline planes of the X-rays and the intensities of these rays. In order for the detector to collect all curvilinear rays, the detector moves around the axis of the shape on a circle where the angles and corners of the vertices are generated by a two-dimensional plan called the X-ray diagram. This diagram is characteristic for the material (fingerprint).

Determination the particles size of the silver nanoparticle from Debye – Scherrer's Equation[12]:

The broadening of the Bragg reflection peaks indicates that the crystallite domain size is in nanosizes. The broadening at half maximum intensity of the diffraction peak is related to a reduction in crystallite size, flattening and micro-strands within the diffraction domains. The average particle size D was determined by using Scherrer's formula:

$$D = \frac{K.\lambda}{\beta.\cos\theta}$$

β is the full width at half maximum (FWHM), θ is the diffraction angle, D is the average crystallite size perpendicular to the reflecting planes and λ is the X-ray wavelength, k is constant, its value is close to one, and is related to crystalline shapes and lattice plane which corresponding to studied peak, if we assume that the shape is spherical, k will be 0.9. The FWHM for each sample were taken from the (111) Bragg's reflection and its value were estimated by fitting the peaks using origin program.

Table S4. Calculation of diffraction angle, FWHM and crystallite sizes for sample 16 at different times and some other samples of biosynthesized silver nanoparticles.

sample number	2θ by degree	β FWHM (rad)	Size of the particle (D) nm
16	38.214	0.0057	25.6
16	38.230	0.0054	27.4
16	38.207	0.0061	24
16	37.796	0.015	10

16	38.207	0.0107	15
15	38.222	0.0072	20.3
17	37.939	0.0105	14
18	38.324	0.0069	21.4

The calculated crystallite size of as-prepared Ag-NPs (~ 24-27 nm) for sample 16 at some times are compatible with the values obtained from SEM and TEM apparatus. The crystallite size of Ag-NPs for some others of sample 16 (~10-15 nm) is rather smaller compared to the average nanoparticle size estimated from SEM and TEM observation (~25-40 nm), suggesting that there are a small nanocrystals in the multiple twined silver nanoparticles.

Probable mechanism of biosynthesis of silver nanoparticles by plant extracts:

Table S5. plant components of plant material extract which act as a capping and reducing agent during green synthesis of AgNPs from different plant species.

Plant genus	Metabolites found in the Extract/AgNPs	Reference
Azadirachta indica	Flavanoids, terpenoids	[13]
Hybanthus enneaspermus	Several bioactive phytochemical compounds	[14]
Syzygium cumini	Flavonoids	[15]
Ocimum sanctum	Flavonoid, proteins, gallic acid, terpenoids	[16]
Nigella arvensis	Flavonoids, alkaloids	[17]
Coleus aromaticus	Flavonoids	[18]
Glycyrrhiza glabra	Flavonoids, thiamine and terpenoids	[19]
Lantana camara	Flavonoids, glycosides and carbohydrates	[20]
Rosmarinus officinalis	Polyphenols	[21]
Citrus sinensis	Flavonoids, ascorbic acid, volatile oils	[22]
Mimusops elengi	Polyphenols	[23]
Dioscorea bulbifera	Flavonoids, polyphenols	[24]
Syzygium cumini	Polyphenols	[25]
Decalepis hamiltonii	Polyols, phenols	[26]
Plumeria rubra	Proteins	[27]
Gardenia jasminoides	Rutin, gallic acid, chlorogenic acid	[28]
Helianthus annuus	Flavonoids, proteins, amino acids, amides terpenoids	[29]
Boswellia serrata	Proteins	[30]
Lippia citriodora	Verbascoside, isoverbascoside, chrysoeriol-7-O-diglucoronide, luteonin-7-O-diglucoronide	[31]
Solanum xanthocarpum	Alkaloids, phenolics, sugars	[32]
Withania somnifera	Catechin p-coumaric acid, luteolin-7-glucoside, withanolides	[33]
Achyranthes aspera	Polyols	[34]
Ocimum sanctum	Quercetin	[35]
Leonuri herba	Hydroxyl, polyphenols groups	[36]
Desmodium triflorum	Ascorbic acid	[37]
Trianthema decandra	Saponin	[38]
Mentha piperita	Alkaloids, flavones, steroids, polysaccharides, amino acids,	[39]

	Oximes, proteins, menthol	
Anacardium occidentale	Proteins, polyols	[40]
Zingiber officinale	Flavonoid, alkaloids	[41]
Alternanthera sessilis	Tannins, carbohydrates, proteins, ascorbic acid	[42]
Dioscorea bulbifera	Diosgenin, ascorbic acid	[24]
Morinda pubescens	Catechins, hydroxyflavones	[43]
Annona squamosa	Alkaloids, glycoside, saponins, tannins,	[44]
	Phenolic compounds, carbohydrates	
Hibiscus rosa- sinensis	Carboxylate ion groups	[45]
Acalypha indica	Quercetin, plant pigment	[46]
Carica papaya	Catechnis, hydroxyflavones	[47]
Eucalyptus	Alcohol, phenols, alkylaldehyde	[48]
Aegle marmelos	Tannin	[49]
Mangifera indica	Ketone, aldehydes, hydroxyl, carboxyl groups	[50]
Lonicera japonica	Phenolic and hydroxyl groups of chlorogenic acid	[51]

References:

1. Paramelle, D.; Sadovoy, A.; Gorelik, S.; Free, P.; Hobley, J.; Fernig, D.G. A rapid method to estimate the concentration of citrate capped silver nanoparticles from UV-visible light spectra. *Analyst* **2014**, *139*, 4855-4861, doi:10.1039/c4an00978a.
2. Pavia, D.L.; Lampman, G.M.; Kriz, G.S.; Vyvyan, J.A. *Introduction to spectroscopy*; Cengage Learning: 2008.
3. Nichita, C.; Giurginca, M.; Bazdoaca, C.; Parvu, L.; Meghea, A. Vegetal antioxidants obtained from Hyperycum perforatum species. *Revista De Chimie* **2007**, *58*, 910-913.
4. Ashokkumar, R.; Ramaswamy, M. Phytochemical screening by FTIR spectroscopic analysis of leaf extracts of selected Indian Medicinal plants. *Int J Curr Microbiol Appl Sci* **2014**, *3*, 395-406.
5. Kozarski, M.; Klaus, A.; Vunduk, J.; Zizak, Z.; Niksic, M.; Jakovljevic, D.; Vrvic, M.M.; Van Griensven, L.J. Nutraceutical properties of the methanolic extract of edible mushroom Cantharellus cibarius (Fries): primary mechanisms. *Food Funct* **2015**, *6*, 1875-1886, doi:10.1039/c5fo00312a.
6. Williams, D.B.; Carter, C.B.; Veyssiere, P. *Transmission electron microscopy: a textbook for materials science*; Springer: 1998; Vol. 10.
7. Prokofiev, E.A.; Burow, J.A.; Payton, E.J.; Zarnetta, R.; Frenzel, J.; Gunderov, D.V.; Valiev, R.Z.; Eggeler, G. Suppression of Ni4Ti3 Precipitation by Grain Size Refinement in Ni-Rich NiTi Shape Memory Alloys. *Advanced engineering materials* **2010**, *12*, 747-753.
8. Morgagni, F. Instrument.
9. Xu, Z.W.; Ngan, A.H.W. TEM study of electron beam-induced crystallization of amorphous GeSi films. *Philosophical Magazine Letters* **2004**, *84*, 719-728, doi:10.1080/14786430500038088.
10. Mogilevsky, P.; Hay, R.S.; Boakye, E.E.; Keller, K.A. Evolution of texture in rhabdophane-derived monazite coatings. *Journal of the American Ceramic Society* **2003**, *86*, 1767-1772, doi:DOI 10.1111/j.1151-2916.2003.tb03552.x.
11. Egerton, R.F. *Physical principles of electron microscopy*; Springer: 2005.
12. Theivasanthi, T.; Alagar, M. Electrolytic synthesis and characterizations of silver nanopowder. *arXiv preprint arXiv:1111.0260* **2011**.
13. Ahmed, S.; Saifullah; Ahmad, M.; Swami, B.L.; Ikram, S. Green synthesis of silver nanoparticles using Azadirachta indica aqueous leaf extract. *J Radiat Res Appl Sc* **2016**, *9*, 1-7, doi:10.1016/j.jrras.2015.06.006.
14. Suman, T.Y.; Rajasree, S.R.R.; Jayaseelan, C.; Mary, R.R.; Gayathri, S.; Aranganathan, L.; Remya, R.R. GC-MS analysis of bioactive components and biosynthesis of silver nanoparticles using Hybanthus enneaspermus at room temperature evaluation of their stability and its larvicidal activity. *Environmental Science and Pollution Research* **2016**, *23*, 2705-2714, doi:10.1007/s11356-015-5468-5.
15. Prasad, R.; Swamy, V.S. Antibacterial activity of silver nanoparticles synthesized by bark extract of Syzygium cumini. *Journal of Nanoparticles* **2013**, *2013*.
16. Ramteke, C.; Chakrabarti, T.; Sarangi, B.K.; Pandey, R.-A. Synthesis of silver nanoparticles from the aqueous extract of leaves of Ocimum sanctum for enhanced antibacterial activity. *Journal of chemistry* **2012**, *2013*.
17. Chahardoli, A.; Karimi, N.; Fattahi, A. Biosynthesis, Characterization, Antimicrobial and Cytotoxic Effects of Silver Nanoparticles Using Nigella arvensis Seed Extract. *Iranian Journal of Pharmaceutical Research* **2017**, *16*, 1167-1175.
18. Vanaja, M.; Annadurai, G. Coleus aromaticus leaf extract mediated synthesis of silver nanoparticles and its bactericidal activity. *Applied Nanoscience* **2013**, *3*, 217-223, doi:10.1007/s13204-012-0121-9.
19. Dinesh, S.; Karthikeyan, S.; Arumugam, P. Biosynthesis of silver nanoparticles from Glycyrrhiza glabra root extract. *Archives of Applied Science Research* **2012**, *4*, 178-187.

20. Ajitha, B.; Ashok Kumar Reddy, Y.; Shameer, S.; Rajesh, K.M.; Suneetha, Y.; Sreedhara Reddy, P. Lantana camara leaf extract mediated silver nanoparticles: Antibacterial, green catalyst. *J Photochem Photobiol B* **2015**, *149*, 84-92, doi:10.1016/j.jphotobiol.2015.05.020.
21. Ghaedi, M.; Yousefinejad, M.; Safarpoor, M.; Khafri, H.Z.; Purkait, M.K. Rosmarinus officinalis leaf extract mediated green synthesis of silver nanoparticles and investigation of its antimicrobial properties. *Journal of Industrial and Engineering Chemistry* **2015**, *31*, 167-172, doi:10.1016/j.jiec.2015.06.020.
22. Kaviya, S.; Santhanalakshmi, J.; Viswanathan, B.; Muthumary, J.; Srinivasan, K. Biosynthesis of silver nanoparticles using citrus sinensis peel extract and its antibacterial activity. *Spectrochimica Acta Part a-Molecular and Biomolecular Spectroscopy* **2011**, *79*, 594-598, doi:10.1016/j.saa.2011.03.040.
23. Kumar, H.A.K.; Mandal, B.K.; Kumar, K.M.; Maddinedi, S.B.; Kumar, T.S.; Madhiyazhagan, P.; Ghosh, A.R. Antimicrobial and antioxidant activities of Mimusops elengi seed extract mediated isotropic silver nanoparticles. *Spectrochimica Acta Part a-Molecular and Biomolecular Spectroscopy* **2014**, *130*, 13-18, doi:10.1016/j.saa.2014.03.024.
24. Ghosh, S.; Patil, S.; Ahire, M.; Kitture, R.; Kale, S.; Pardesi, K.; Cameotra, S.S.; Bellare, J.; Dhavale, D.D.; Jabgunde, A., et al. Synthesis of silver nanoparticles using Dioscorea bulbifera tuber extract and evaluation of its synergistic potential in combination with antimicrobial agents. *International Journal of Nanomedicine* **2012**, *7*, 483-496, doi:10.2147/Ijn.S24793.
25. Kumar, V.; Yadav, S.C.; Yadav, S.K. Syzygium cumini leaf and seed extract mediated biosynthesis of silver nanoparticles and their characterization. *Journal of Chemical Technology and Biotechnology* **2010**, *85*, 1301-1309, doi:10.1002/jctb.2427.
26. Rashmi, V.; Sanjay, K.R. Green synthesis, characterisation and bioactivity of plant-mediated silver nanoparticles using Decalepis hamiltonii root extract. *IET nanobiotechnology* **2016**, *11*, 247-254.
27. Patil, C.D.; Patil, S.V.; Borase, H.P.; Salunke, B.K.; Salunkhe, R.B. Larvicidal activity of silver nanoparticles synthesized using Plumeria rubra plant latex against Aedes aegypti and Anopheles stephensi. *Parasitology Research* **2012**, *110*, 1815-1822, doi:10.1007/s00436-011-2704-x.
28. Fenfen, L.; Yixian, G.; HUANG, J.; Daohua, S.; Qingbiao, L. Roles of biomolecules in the biosynthesis of silver nanoparticles: case of Gardenia jasminoides extract. *Chinese Journal of Chemical Engineering* **2014**, *22*, 706-712.
29. Thakore, S.; Rathore, P.S.; Jadeja, R.N.; Thounaojam, M.; Devkar, R.V. Sunflower oil mediated biomimetic synthesis and cytotoxicity of monodisperse hexagonal silver nanoparticles. *Mater Sci Eng C Mater Biol Appl* **2014**, *44*, 209-215, doi:10.1016/j.msec.2014.08.019.
30. Kora, A.J.; Sashidhar, R.B.; Arunachalam, J. Aqueous extract of gum olibanum (Boswellia serrata): A reductant and stabilizer for the biosynthesis of antibacterial silver nanoparticles. *Process Biochemistry* **2012**, *47*, 1516-1520, doi:10.1016/j.procbio.2012.06.004.
31. Cruz, D.; Fale, P.L.; Mourato, A.; Vaz, P.D.; Serralheiro, M.L.; Lino, A.R. Preparation and physicochemical characterization of Ag nanoparticles biosynthesized by Lippia citriodora (Lemon Verbena). *Colloids Surf B Biointerfaces* **2010**, *81*, 67-73, doi:10.1016/j.colsurfb.2010.06.025.
32. Sengottaiyan, A.; Mythili, R.; Selvankumar, T.; Aravinthan, A.; Kamala-Kannan, S.; Manoharan, K.; Thiyagarajan, P.; Govarthanan, M.; Kim, J.H. Green synthesis of silver nanoparticles using Solanum indicum L. and their antibacterial, splenocyte cytotoxic potentials. *Research on Chemical Intermediates* **2016**, *42*, 3095-3103, doi:10.1007/s11164-015-2199-7.
33. Marslin, G.; Selvakesavan, R.K.; Franklin, G.; Sarmento, B.; Dias, A.C. Antimicrobial activity of cream incorporated with silver nanoparticles biosynthesized from Withania somnifera. *Int J Nanomedicine* **2015**, *10*, 5955-5963, doi:10.2147/IJN.S81271.
34. Elumalai, D.; Kaleena, P.; Ashok, K.; Suresh, A.; Hemavathi, M. Green synthesis of silver nanoparticle using Achyranthes aspera and its larvicidal activity against three major mosquito vectors. *Engineering in Agriculture, Environment and Food* **2016**, *9*, 1-8.
35. Jain, S.; Mehata, M.S. Medicinal plant leaf extract and pure flavonoid mediated green synthesis of silver nanoparticles and their enhanced antibacterial property. *Scientific reports* **2017**, *7*, 15867.
36. Im, A.R.; Han, L.; Kim, E.R.; Kim, J.; Kim, Y.S.; Park, Y. Enhanced antibacterial activities of leonuri herba extracts containing silver nanoparticles. *Phytother Res* **2012**, *26*, 1249-1255, doi:10.1002/ptr.3683.
37. Ahmad, N.; Sharma, S.; Singh, V.N.; Shamsi, S.F.; Fatma, A.; Mehta, B.R. Biosynthesis of Silver Nanoparticles from Desmodium triflorum: A Novel Approach Towards Weed Utilization. *Biotechnol Res Int* **2011**, *2011*, 454090, doi:10.4061/2011/454090.
38. Geethalakshmi, R.; Sarada, D.V.L. Characterization and antimicrobial activity of gold and silver nanoparticles synthesized using saponin isolated from Trianthema decandra L. *Industrial Crops and Products* **2013**, *51*, 107-115, doi:10.1016/j.indcrop.2013.08.055.
39. MubarakAli, D.; Thajuddin, N.; Jeganathan, K.; Gunasekaran, M. Plant extract mediated synthesis of silver and gold nanoparticles and its antibacterial activity against clinically isolated pathogens. *Colloids Surf B Biointerfaces* **2011**, *85*, 360-365, doi:10.1016/j.colsurfb.2011.03.009.
40. Mukunthan, K.; Balaji, S. Cashew apple juice (Anacardium occidentale L.) speeds up the synthesis of silver nanoparticles. *International Journal of Green Nanotechnology* **2012**, *4*, 71-79.
41. Velmurugan, P.; Anbalagan, K.; Manosathyadevan, M.; Lee, K.J.; Cho, M.; Lee, S.M.; Park, J.H.; Oh, S.G.; Bang, K.S.; Oh, B.T. Green synthesis of silver and gold nanoparticles using Zingiber officinale root extract and antibacterial activity of silver nanoparticles against food pathogens. *Bioprocess and Biosystems Engineering* **2014**, *37*, 1935-1943, doi:10.1007/s00449-014-1169-6.
42. Niraimathi, K.L.; Sudha, V.; Lavanya, R.; Brindha, P. Biosynthesis of silver nanoparticles using Alternanthera sessilis (Linn.) extract and their antimicrobial, antioxidant activities. *Colloids and Surfaces B-Biointerfaces* **2013**, *102*, 288-291, doi:10.1016/j.colsurfb.2012.08.041.

43. Kumar, K.R.; Nattuthurai, N.; Gopinath, P.; Mariappan, T. Synthesis of eco-friendly silver nanoparticles from Morinda tinctoria leaf extract and its larvicidal activity against Culex quinquefasciatus. *Parasitol Res* **2015**, *114*, 411-417, doi:10.1007/s00436-014-4198-9.
44. Vivek, R.; Thangam, R.; Muthuchelian, K.; Gunasekaran, P.; Kaveri, K.; Kannan, S. Green biosynthesis of silver nanoparticles from Annona squamosa leaf extract and its in vitro cytotoxic effect on MCF-7 cells. *Process Biochemistry* **2012**, *47*, 2405-2410, doi:10.1016/j.procbio.2012.09.025.
45. Nayak, D.; Ashe, S.; Rauta, P.R.; Nayak, B. Biosynthesis, characterisation and antimicrobial activity of silver nanoparticles using Hibiscus rosa-sinensis petals extracts. *Iet Nanobiotechnology* **2015**, *9*, 288-293, doi:10.1049/iet-nbt.2014.0047.
46. Krishnaraj, C.; Jagan, E.G.; Rajasekar, S.; Selvakumar, P.; Kalaichelvan, P.T.; Mohan, N. Synthesis of silver nanoparticles using Acalypha indica leaf extracts and its antibacterial activity against water borne pathogens. *Colloids and Surfaces B-Biointerfaces* **2010**, *76*, 50-56, doi:10.1016/j.colsurfb.2009.10.008.
47. Banala, R.R.; Nagati, V.B.; Karnati, P.R. Green synthesis and characterization of Carica papaya leaf extract coated silver nanoparticles through X-ray diffraction, electron microscopy and evaluation of bactericidal properties. *Saudi J Biol Sci* **2015**, *22*, 637-644, doi:10.1016/j.sjbs.2015.01.007.
48. Liu, Y.; Jin, X.Y.; Chen, Z.L. The formation of iron nanoparticles by Eucalyptus leaf extract and used to remove Cr(VI). *Science of the Total Environment* **2018**, *627*, 470-479, doi:10.1016/j.scitotenv.2018.01.241.
49. Rao, K.J.; Paria, S. Green synthesis of silver nanoparticles from aqueous Aegle marmelos leaf extract. *Materials Research Bulletin* **2013**, *48*, 628-634, doi:10.1016/j.materresbull.2012.11.035.
50. Yang, N.; Li, W.H. Mango peel extract mediated novel route for synthesis of silver nanoparticles and antibacterial application of silver nanoparticles loaded onto non-woven fabrics. *Industrial Crops and Products* **2013**, *48*, 81-88, doi:10.1016/j.indcrop.2013.04.001.
51. Zhou, Y.Y.; Tang, R.C. Facile and eco-friendly fabrication of AgNPs coated silk for antibacterial and antioxidant textiles using honeysuckle extract. *Journal of Photochemistry and Photobiology B-Biology* **2018**, *178*, 463-471, doi:10.1016/j.jphotobiol.2017.12.003.
